# Predicting Risk of Long‐Term Institutionalization Among Community Dwelling Veterans Before the COVID‐19 Pandemic

**DOI:** 10.1111/1475-6773.70016

**Published:** 2025-07-26

**Authors:** Bruce Kinosian, Susan Schmitt, Matthew Augustine, Scotte Hartronft, Rajesh Makineni, Kimberly Judon, Gregory Krautner, Cheryl Schmitz, Mary K. Goldstein, Ciaran S. Phibbs, Orna Intrator

**Affiliations:** ^1^ Geriatrics and Extended Care Data Analysis Center (GECDAC) Bronx New York USA; ^2^ Cpl Michael J Crescenz VA Medical Center Philadelphia Pennsylvania USA; ^3^ Leonard Davis Institute for Health Economics, University of Pennsylvania Philadelphia Pennsylvania USA; ^4^ Department of Medicine, Division of Geriatrics Perelman School of Medicine, University of Pennsylvania Philadelphia Pennsylvania USA; ^5^ VA Palo Alto Health Care System Palo Alto California USA; ^6^ Department of Pediatrics Stanford University Palo Alto California USA; ^7^ James J Peters VAMC Bronx New York USA; ^8^ Department of Geriatrics & Palliative Care Icahn School of Medicine at Mount Sinai New York New York USA; ^9^ Department of Veterans Affairs Geriatrics and Extended Care Washington DC USA; ^10^ Department of Public Health Sciences University of Rochester Rochester New York USA; ^11^ Department of Health Policy Stanford University Palo Alto California USA; ^12^ VA Health Systems Research Center for Innovation to Implementation (Ci2i), VA Palo Alto Health Care System Palo Alto California USA

**Keywords:** cost, HCBS veterans, hospitalization, mortality, VA

## Abstract

**Objective:**

To identify risk of long‐term institutionalization (LTI) among Veterans receiving care in the Veterans Health Administration (VA).

**Study Setting and Design:**

We developed the “Predicted Long‐term Institutionalization” (PLI) risk model for Veterans alive in the community at the end of fiscal‐year (FY) 2017 followed for LTI in nursing home (cumulative NH days allowing any acute care and up to 7 days in community > 90 days) during FY2018‐FY2019.

**Data Sources and Analytic Sample:**

PLI used demographics, diagnoses, prior hospital and nursing home (NH) use, and risk indices for death and frailty from VA and Medicare claims and Minimum Data Set data. Development of PLI used multiple iterations to maximize sensitivity, constrained by achieving a number needed to screen (≤ 8), including age normalization to minimize algorithmic bias. We combined the elevated risk (ER) and common risk (CR) strata‐specific predictions from the logistic regression models to identify three tiers of PLI: low risk, moderate risk, and high risk. We describe Veterans' outcomes in FY2018/2019 (LTI, death, hospitalization and VA cost) across the three PLI tiers.

**Principal Findings:**

For identifying Veterans in LTI, compared to a baseline model that used only VA data as predictors (sensitivity 23%, specificity 98%), calibrating separate ER and CR strata increased sensitivity to 30%, the addition of Medicare data increased sensitivity to 33%, and age‐normalization with differential risk strata thresholds increased sensitivity to 41% (specificity 96.6%). The final PLI model (c‐statistic = 0.87) identified 3.5% of Veterans in PLI‐high risk (13% LTI rate), who accounted for 41% of new LTI, 22% of decedents, 19% of VA cost, and 11% of hospitalizations in FY2018–2019.

**Conclusions:**

The PLI score identifies Veterans at high risk of LTI for further assessment and targeting of resources to support continued community residence.

**Summary**

- What is known on this topic
  ○ Deployment of limited home and community‐based services (HCBS) to those at greatest risk of long‐term institutionalization (LTI) can delay LTI, supporting Veterans' desires to remain in the community.
  ○ LTI within 2 years among Veterans living in the community is 1.1%.
  ○ Identifying individuals at high risk of LTI from population‐based administrative data is challenging due to the absence of information on key drivers of LTI.
- What this study adds
  ○ Modeling LTI improves targeting HCBS by accounting for data lags in Medicare claims data, incorporating a claims frailty index, and calibrating separate models for elevated and common risk segments of the population.
  ○ Combining VA and Medicare claims data, the PLI can identify 41% of Veterans cared for in the VA who experience LTI in the next 2 years.
  ○ Identifying Veterans at highest risk of future LTI can optimize the value of home‐ and community‐based services to meet Veteran needs.

## 1 Introduction

Unlike most of the US health care system, the Veterans Health Administration (VA) is responsible for long‐term services and supports, in addition to physical and behavioral health. The rising demand for nursing home care due to an aging population may be lower in the VA since home‐ and community‐based services (HCBS), designed to make it possible for patients to remain in the community, are available to all Veterans as a basic benefit. These services are otherwise only available with government insurance to those who are on Medicaid. By 2037, the number of Veterans over the age of 85 is expected to rise by 70% from 435,000 in 2017, while the number of Veterans in community nursing homes paid by VA is expected to double from 9600 [1]. Prior work suggests that in 2019 only 20% of Veterans entering long‐term institutionalization (LTI) had received HCBS [2]. Faced with rising demand for LTI, VHA has instituted a pilot program, Redefining Elder Care in America Program (RECAP), to better align HCBS with Veterans at high risk of LTI. If more appropriate Veterans receive HCBS, more future NH care may be delayed or limited [3, 4, 5]. RECAP implements an integrated care management system using traditional clinical referrals, a population health approach for proactively identifying Veterans at high risk of LTI and conducting comprehensive geriatric assessment to high‐risk Veterans to determine which services can best mitigate their LTI risk and enhance their independence [6]. Identifying Veterans at high risk of LTI is crucial for RECAP efficiency.

Predicting LTI risk from healthcare claims is challenging. Among VA users, LTI has a low prevalence (1%), while five times as many Veterans identified at high risk of LTI die before entering a nursing home [2]. Based on prior literature, information predictive of LTI risk includes modifiable factors (e.g., unmet functional needs, resilience) and non‐modifiable factors (e.g., family supports, dementia progression). Much of this information is not routinely available in population‐level administrative data [7, 8, 9, 10], although recent development of claims‐based frailty models suggests that diagnostic information can identify those with functional dependencies [11, 12, 13].

To improve the effectiveness of RECAP, our objective was to improve an existing VA LTI risk model, which was deployed in an earlier effort to target home and community‐based services (Choose Home [2]) to develop the PLI (Predicted Long‐term Institutionalization) measure using national VHA and non‐VHA data, reflecting a bedside‐to‐model pipeline [14]. Novel development strategies included incorporating a diagnosis‐based frailty index (the JEN Frailty Index (JFI)) [15], a VA‐developed hospitalization and mortality risk index (the Care Assessment Needs (CAN) [16] score) and a population risk stratification strategy to enable better model calibration. Since many Veterans use Medicare‐paid services in addition to VA care, we incorporated information from Traditional Medicare claims into PLI, expanding beyond the limitations of current VA‐based risk measures [16]. Moreover, since long‐term care is often not paid by Medicare or VA, we included data from nursing home resident assessments (Minimum Data Set, MDS) to ascertain LTI. Together, these methods combined to improve the ability to identify Veterans at risk of LTI. The final model is the new measure, PLI, which can be used to operationally identify high‐risk Veterans to target HCBS.

## 2 Methods

### 2.1 Study Population

From all Veterans receiving care in the VHA in FY2017 (*n* = 6,076,071), we excluded Veterans who were identified as being institutionalized long‐term or receiving hospice care in FY2017 (*N* = 63,139), those without a face‐to‐face utilization (*n* = 215,059), Veterans who died in FY2017 (*n* = 143,609), and those in a hospital or nursing home on the last day of 2017 (*N* = 187,048; Figure S1). We required diagnoses only from face‐to‐face utilizations, as CMS does for risk scoring, due to their greater accuracy for diagnostic data.

### 2.2 Data Sources

Covariate information was primarily from fiscal year (FY) 2017. We used VA inpatient, outpatient, and enrollment records, VA‐purchased care claims records from FEE and the Program Integrity Tool, Medicare enrollment, and Traditional Medicare claims data to retrieve diagnoses, utilization, costs, and demographic information. Cost data were retrieved from the VHA Managerial Cost Accounting Decision Support System, Fee‐basis, and Medicare reimbursements. VA provided utilization records; VA purchased care claims; and Traditional Medicare claims, along with NH resident assessment Minimum Data Set (MDS) [17] data from the Centers of Medicare and Medicaid Services (CMS), VA Community Living Centers (CLCs are VA owned and operated nursing homes), and State Veteran Home nursing homes were used to create the VA Residential History File (RHF) [18]. The RHF tracks daily location and health care services use. MDS assessment dates and times are used in the RHF to track days that are likely in nursing home regardless of payer, covering all but 253 non‐certified NHs in the U.S. Thus, the RHF can be used to identify practically all LTI.

### 2.3 Study Outcomes

The primary outcome was any LTI during FY2018 and FY2019. LTI was defined as more than 90 consecutive days of any (VA or non‐VA) nursing home (NH) care. Consecutive NH days were allowed to be interrupted by any number of acute hospital or emergency department days but with no more than 7 consecutive days in the community. Two‐year LTI as the target, used in prior VA HCBS efforts, provides sufficient time to provide services that can effectuate change [2].

Secondary study outcomes include death in FY2018–FY2019, total VA cost of care in FY2018, any use of personal care services (home‐maker or home‐health aide, respite, adult day health care, Veteran‐Directed Care, and Program for All‐inclusive Care of the Elderly) in FY2018, and any use of non‐institutional Geriatric and Extended Care services in FY2018 (e.g., geriatric primary care through the Geriatric Patient‐Aligned Care Teams, community‐based palliative care and hospice consults, Home‐Based Primary Care, and purchased skilled nursing care for nursing and therapies).

### 2.4 Covariates and Stratification Variables

We created diagnosis‐based risk variables and comorbidity clusters to capture patient‐level characteristics associated with increased risk of 2‐year LTI. Conditions with no identifying diagnoses found in the data were assumed to not be present, potentially biasing estimates to the null.

Variables used in the models for LTI prediction were identified in the original *Choose Home* model [2], in other literature, or by Geriatric and Extended Care clinical leaders. Additional variables included socio‐demographic and enrollment information, prior year utilization (any LTI, any Medicare‐paid skilled nursing facility (SNF) care in FY2016, and any hospitalizations in FY2017), risk scores (JFI, CAN), and FY2017 total VA cost of care from VA's Decision Support System and Fee‐basis. Cost data were cleaned, outlier corrected, and adjusted to geographic differences using the area wage index at the parent facility by VA's Health Economics Resource Center [19] (Table 1 includes all variables used in the PLI model).

**TABLE 1:** Prevalence and average marginal effects (AME) on long‐term institutionalization (LTI) of demographics, risk scores, health care use, and diagnoses of Veteran populations in the elevated risk (ER) and common‐risk (CR) strata.

Characteristic	Elevated risk (ER)^a^						Common risk (CR)				
	*N* = 507,304						*N* = 4937,032				
	*N*/mean	% within/SD	% of total in ER	AME	LCI	UCI	*N*	% within/SD	AME	LCI	UCI
Demographics											
Age	70.87	12.26		−0.001	−0.002	−0.001	60.37	16.47	0.000	−0.001	0.000
Male gender	483,461	95.3%	9.7%	0.002	−0.001	0.005	4,502,590	91.2%	0.001	0.000	0.001
Married	312,499	61.6%	8.4%	−0.016	−0.017	−0.015	3,411,502	69.1%	−0.003	−0.003	−0.003
Rural	127,841	25.2%	7.2%	−0.011	−0.013	−0.010	1,644,038	33.3%	−0.003	−0.003	−0.002
VA priority Group 1^a^	216,619	42.7%	9.1%	−0.003	−0.004	−0.001	2,157,491	43.7%	−0.001	−0.001	−0.001
Homeless	24,351	4.8%	16.5%	0.016	0.014	0.019	123,426	2.5%	0.004	0.004	0.004
Risk scores											
JFI	7.78	1.66		0.004	0.003	0.004	2.92	1.89	0.000	0.000	0.000
CAN	30.5%	23%		0.015	0.012	0.018	8.8%	9.1%	0.010	0.010	0.011
CAN, missing	18,263	3.6%		0.035	0.032	0.038	222,167	4.5%	0.004	0.004	0.004
Health care use in prior year											
Hospitalization	346,489	68.3%	59.9%	0.008	0.007	0.010	232,041	4.7%	−0.001	−0.002	−0.001
SNF stay	76,096	15.0%	72.0%	0.014	0.013	0.015	29,622	0.6%	0.005	0.005	0.006
Prior LTI	4566	0.9%	48%	0.035	0.032	0.039	4937	0.1%	0.007	0.006	0.007
VA total costs	$38,410	$6130	34.2%	0.001	0.001	0.001	$7610	$16,520	0.000	0.000	0.000
Diagnoses in prior year											
Amputation	18,263	3.6%	48%	0.012	0.009	0.014	19,748	0.4%	0.004	0.004	0.005
Cancer	130,377	25.7%	24.6%	−0.012	−0.014	−0.011	399,901	8.1%	−0.002	−0.002	−0.002
Dementia	77,110	15.2%	38.5%	0.039	0.037	0.040	123,426	2.5%	0.009	0.009	0.009
Diabetes	253,652	50.0%	17.2%	0.006	0.005	0.008	1,224,388	24.8%	0.001	0.001	0.001
Fracture	20,292	4.0%	57.8%	0.013	0.011	0.016	14,811	0.3%	0.002	0.001	0.003
Head injury	25,873	5.1%	12.1%	−0.008	−0.011	−0.005	187,608	3.8%	−0.002	−0.003	−0.002
Heart failure	173,498	34.2%	42.3%	0.000	−0.002	0.001	236,978	4.8%	0.000	0.000	0.000
Malnutrition	37,033	7.3%	71.4%	0.007	0.006	0.009	14,833	0.3%	0.003	0.002	0.003
Mult. Sclerosis	4058	0.8%	21.5%	0.020	0.014	0.025	14,811	0.3%	0.006	0.005	0.007
Obesity	143,567	28.3%	15.5%	−0.001	−0.002	0.001	784,991	15.9%	−0.001	−0.001	−0.001
Parkinson's	19,785	3.9%	30.8%	0.021	0.019	0.023	44,433	0.9%	0.006	0.006	0.007
Pressure ulcer	58,487	11.6%	52.0%	0.018	0.017	0.020	54,308	1.1%	0.004	0.004	0.004
Schizophrenia	17,248	3.4%	18.9%	0.027	0.025	0.030	74,056	1.5%	0.007	0.006	0.007
Spinal cord Inj.	20,799	4.1%	45.7%	0.007	0.005	0.010	24,685	0.5%	0.004	0.003	0.004
Seizure	34,497	6.8%	33.3%	0.008	0.006	0.010	69,119	1.4%	0.003	0.002	0.003
Sepsis	63,850	12.6%	75.5%	−0.001	−0.002	0.001	20,664	0.42%	0.001	0.001	0.002
Stroke	74,574	14.7%	44.3%	0.018	0.016	0.019	93,804	1.9%	0.004	0.004	0.004
Subst. Use Dis.	94,866	18.7%	17.8%	0.004	0.002	0.006	439,397	8.9%	0.001	0.001	0.001

*Note:* Study population includes 5,444,354 veterans using VA in FY2017 and alive on the last day of FY2017, excluding those with LTI, in hospital, or nursing home on the last day of FY2017, and those who did not have any face‐to‐face diagnoses in FY 2017. Elevated Risk stratum includes any veterans meeting Independence‐at‐Home qualification (IAH‐Q) on any day of FY2017 or High‐Need/High‐Risk (HNHR) indicator in any month of FY 2017. CR stratum did not meet these criteria.

Abbreviations: AME, average marginal effect; LCI, lower 95% confidence interval; OR, odds ratio; SD, standard deviation; SNF, skilled nursing facility; UCI, upper 95% confidence interval.

^a^ Priority Group 1 are veterans with 70% or higher service‐connected disabilities.

The Veteran's home facility was identified as the VA parent facility with the most days providing care (in case of ties, the most recent facility) [20].

Independence‐at‐Home qualification (IAH‐Q) was adopted from the CMS demonstration and defined by having an acute hospitalization and post‐acute care in the prior year, two or more chronic conditions, and two or more Activities of Daily Living (ADL) limitations. Since we did not have population‐wide ADL measures, our IAH‐Q criteria instead used a JFI score ≥ 6 [21]. The JFI is a diagnosis‐based measure of frailty calibrated on concurrent ADL impairments and future LTI, with a JFI score ≥ 6 equivalent to the 2+ ADL criterion [15]. The High‐Need/High‐Risk (HNHR) indicator is an alternative to IAH‐Q using only VA data that is used to identify Veterans in need of home‐based primary care (HBPC) by relaxing the post‐acute care requirement. HNHR identifies Veterans who within one year prior to an index date had at least one VA or purchased‐care hospitalization, were not in hospice or palliative care and not referred to them, were not in HBPC or Community Living Center. HNHR requires a JFI score (using VA data alone) of 5 or above. HNHR also excludes Veterans identified with end‐stage renal disease. Monthly IAH‐Q and HNHR were calculated during FY2017, with the final model defining status as any month in the prior year with an IAH‐Q or HNHR indicator.

We used the Care Assessment Needs (CAN) score, a VA risk measure of hospitalization and death, to ascertain the predicted probabilities of 1‐year death or hospitalization closest to and prior to the prediction frame target date, October 1, 2017 [16]. Using only VA data, CAN provides weekly scores obtained from demographics, diagnoses, utilization, and select clinical data to predict the risk of either death, hospitalization, or both within the next year. Due to the CAN production rules (e.g., produced only to Veterans assigned to Patient Aligned Care Teams), some Veterans were missing CAN scores and were identified by an indicator of “missing CAN” Of note, CAN has been shown to underpredict mortality risk among Black veterans due to a racially differential pattern of age at death, which can be mitigated by using a race‐centered age measure [22, 23]. Race‐centered age is the residual of a Veteran's age from the average age across Veterans of the same racial‐ethnic group. Finding a similar downward bias on LTI risk among Black Veterans (indicated by a higher LTI false negative rate) with more LTI occurring at younger ages among Black Veterans (Figure S2), we used the race‐centered age approach to mitigate the LTI bias in our final model.

### 2.5 Modeling Strategy

The PLI measure was developed in stages, with each stage adding data sources, variables, and stratification to calculate the incremental value of each stage beyond the previously published VA model (stages presented in Data S1—Model Development) [2]. Model 1 improved the identification of LTI (by using the RHF and including Medicare and MDS data); Model 2 stratified the data to elevated risk (ER) and common risk (CR) strata. A separate model for LTI was estimated in each stratum using logistic regression. Model 3 added prior utilization and diagnostic covariates; Model 4 added JFI; Model 5 added Medicare data to the covariates; Model 6 added station fixed effects; Model 7 allowed Model 6 to have different thresholds for the CR and ER determination of high risk tier. Finally, Model 8 (PLI) changed age to race‐centered age [23] and included only variables that were significant at *p* < 0.05 in one of the prior models. Model 9 repeated Model 8 but used modified covariates to account for delays in updated Medicare data, to be used in the production of real‐time PLI scores for operations.

Regressions were performed using SAS version 9.4. Model performance was evaluated at each step using the area under the curve of the ER and CR models and identifying threshold values for ER and CR models for the high‐risk tier with number needed to screen around 8. Table S1 presents each model with its risk thresholds (for ER and CR strata), number identified in high‐risk tier, number observed LTI among those in the high‐risk tier, sensitivity, specificity, positive predictive value, and number needed to screen of the high‐risk tier in the full data using these thresholds.

**FIGURE 1:**
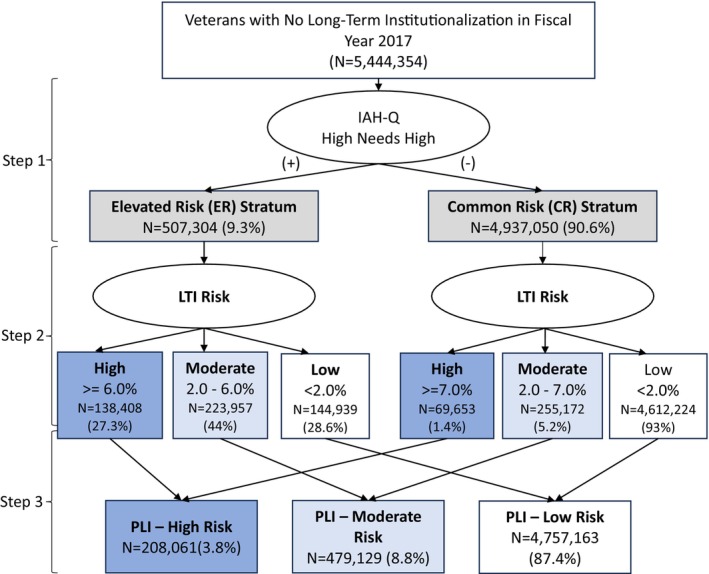
Steps to identify high, moderate, and low tiers of predicting risk of long‐term institutionalization (PLI). Starting from the population of Veterans who used the VA and no long‐term institutionalization in FY 2017, Step 1 stratified veterans into elevated risk (ER) and common risk (CR) if they met or did not meet any Independence‐at‐Home qualification (IAH‐Q) or High Needs High Risk criteria. Step 2 applied predictive models of any LTI for the subsequent 2 years (FY2018‐2019) to each stratum (model 8). Step 3 applied the different risk thresholds in the ER and CR strata to identify high, moderate, and low risk tiers of long‐term institutionalization.

An important step in model development was splitting the study population into two strata of “elevated‐risk” (ER) if they met either Independence‐At‐Home qualification (IAH‐Q) on any day of FY2017 or High‐Need/High‐Risk (HNHR) criteria in any month of FY17 and “common‐risk” (CR) if they did not meet either criterion (Figure 1, Step 1). This step allowed for better calibration by developing different prediction models for each stratum (Step 2) and using different and optimized LTI risk thresholds to identify PLI high risk tier (Step 3), analogous to the first branch of some Random Forest applications [24].

In addition, we accounted for the delays in the receipt of Medicare claims and MDS assessments from CMS. We adjusted Model 8 to compensate for missing data when used for operational screening by extending the look‐back period for Medicare data to 2 years (i.e., through FY16). We called this measure “PLI‐production” (Model 9).

### 2.6 PLI Risk Tiers

The final PLI measure was used to identify a high‐risk tier, using separate risk thresholds for the ER and CR strata. Thresholds were selected to optimally identify the number of Veterans experiencing LTI, while balancing program resource constraints, setting a Number Needed to Screen (NNS) of no more than 8 for one LTI event [25].

We evaluated the model using sensitivity of LTI prediction, the positive predicted value for death or LTI—to compare to the baseline model's outcome, C‐statistics from the logistic regression models to report discrimination, and pseudo r^2^ from the logistic and probit regressions. We created calibration plots to compare the observed to predicted probabilities on predicted probability deciles. Ten‐fold cross‐validation was conducted to validate the model(s) in random subsets of the study cohort, producing a range of C‐statistic values providing a benchmark for comparing different model samples [26].

### 2.7 PLI‐Production Model

The lag in availability of Medicare claims data to VA (typically around 6–9 months) presented challenges to determine the risk of LTI for Veterans incorporating events occurring during the lag period. Since the PLI model assumed full data at the time of risk assessment, we needed to create a production model that accounted for this delay. To compensate for variables based on 12‐month look‐back periods [15, 21, 26], we extended the look‐back period to retrieve covariate information to 24 months, that is, FY2016‐FY2017 (Oct. 1, 2015–Sept. 30, 2017). This included estimating IAH‐Q status based on data from FY 2015 to 2017. For JFI, we used the maximum of the JFI scores each month in FY2017, as JFI is computed based on 12 months look‐back. Since identification of LTI in the prior year required complete CMS data, we identified prior LTI only in months 13–24 prior to the target date (FY2016). We compared the PLI‐production model to the complete data PLI model on prediction performance to assess the impact of the change in data inputs. Thresholds were set with the same constraints as in the complete data PLI measure.

We used the Transparent Reporting of a multivariable prediction model for Individual Prognosis or Diagnosis (TRIPOD) checklist when writing our report [27, 28].

### 2.8 Sensitivity Analyses

The final model (Model 8) was also estimated using probit regression to test the sensitivity of the results to the weight of the left tail (lower probability) of the LTI outcome [29]. We applied the FY2017 model to earlier cohorts (FY2013–2016), each selected using the same criteria as the FY2017 cohort, to determine the out‐of‐sample, inter‐temporal performance to confirm model transportability. We also explicitly compared the logistic model on the 2016–2017 cohort in terms of sensitivity, specificity, and NNS.

## 3 Results

Of the FY2017 cohort (*n* = 5,444,354), 61,875 (1.1%) experienced LTI in FY 2018–2019 and 327,232 (6.1%) died, including 308,958 (5.7%) without any LTI (ratio of 5:1 deaths without LTI to one LTI only event). Nearly 9.3% of Veterans using the VHA met IAH‐Q (6.4%) or HNHR (3.8%) criteria and composed the ER stratum (Figure 1).

Table 1 shows the distribution of Veterans' risk scores, demographic and utilization measures, and relative prevalence of conditions within the ER and CR strata in the final PLI measure (model 8). The ER stratum Veterans were older than the CR stratum Veterans (average age: ER = 71 years, CR = 60 years), more frail (average JFI: ER = 7.8, CR = 2.9), at higher risk of CAN probability of death or hospitalization (mean CAN probability: ER 31%, CR 9%), more costly in the prior year (mean: ER $38,410; CR $7610), more likely to have had prior SNF stays (ER = 15.0%, CR = 0.6%) and to have had one or more acute hospital stays in FY2017 (ER = 68.3%, CR = 4.7%). The prevalence of all conditions was higher in the ER stratum with substantial differences across dementia (ER = 15.2%, CR = 2.5%), malnutrition (ER = 7.3%, CR = 0.3%), and stroke (ER = 14.7%, CR = 1.9%).

### 3.1 PLI Model Development

Final PLI model development used 10 iterations (Table S1). Model 0 was a replication of the original *Choose Home* model (originally developed to predict LTI or death using only VA data) as applied to predict only VA paid LTI and produced a sensitivity of 6.4%, improving, in Model 1, to 23.1% with the addition of Medicare and MDS data to the identification of the LTI outcome. Stratification to ER and CR improved sensitivity to 30.2% (Model 2) and increased to 33.2% with the integration of Medicare data to covariates (Model 5). Adding parent‐station fixed effects increased sensitivity to 34.1% (model 6). The split threshold for LTI risk across ER and CR strata (Model 7) obtained sensitivity of 36.6%.

Model 7 had a different rate of PLI‐High Risk tier membership among Black (2.7%) and White Veterans (3.8%), driven by a higher differential LTI false negative rate for Black Veterans in the CR strata (0.88 vs. 0.77). Using a race‐centered age variable equilibrated the LTI false negative rates in the ER (0.33 vs. 0.34) and CR (0.84 vs. 0.83) strata for Black and White Veterans, respectively. The final algorithmic bias corrected model (Model 8) had a sensitivity of 41.1%, with a NNS of 8.1, making this the second most impactful step on sensitivity after model 2, which introduced stratification to ER and CR strata.

### 3.2 PLI Measure Performance

**FIGURE 2:**
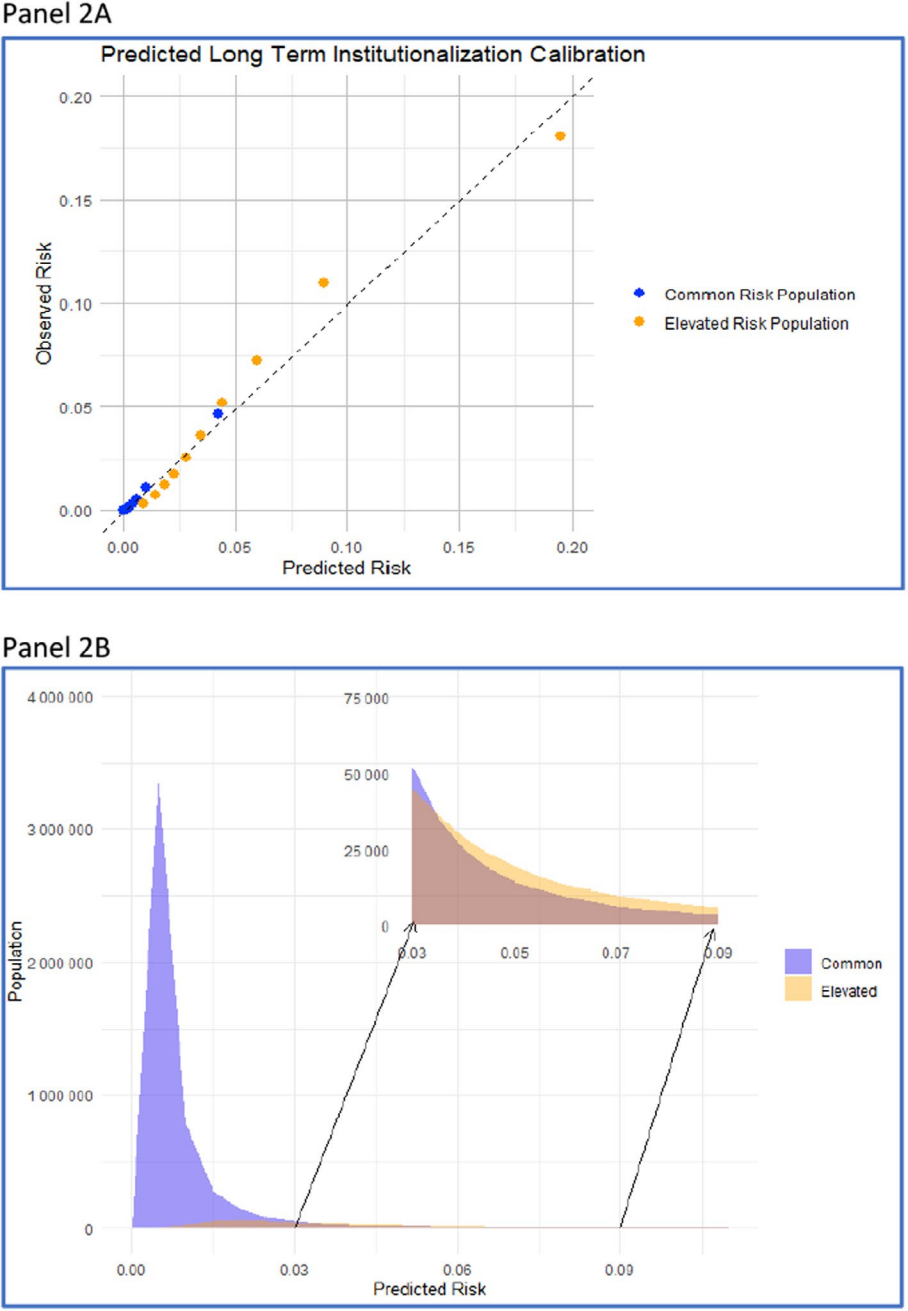
Predicted risk of long‐term Institutionalization (by PLI) and Actual Risk of LTI across Common Risk (CR, blue dots) and Elevated Risk (ER, orange dots) and population distribution (CR, blue, *n* = 5,009,677; ER, beige, *n* = 456,921). For (A) each point represents a 10th of the population's predicted risks.

Calibration plots of the PLI measure (Model 8) presented in Figure 2 show that within the CR stratum the PLI under‐predicted LTI risk by < 1%. Within the ER stratum, the PLI under predicted the risk for the 2nd and 3rd highest deciles by 1.4%–1.8%, but over predicted the risk for the highest decile by 1.5%. Age normalization had minimal effect on calibration, except for the 10th decile of the CR tier, more noticeable among White Veterans (Figures S5 and S6).

**FIGURE 3:**
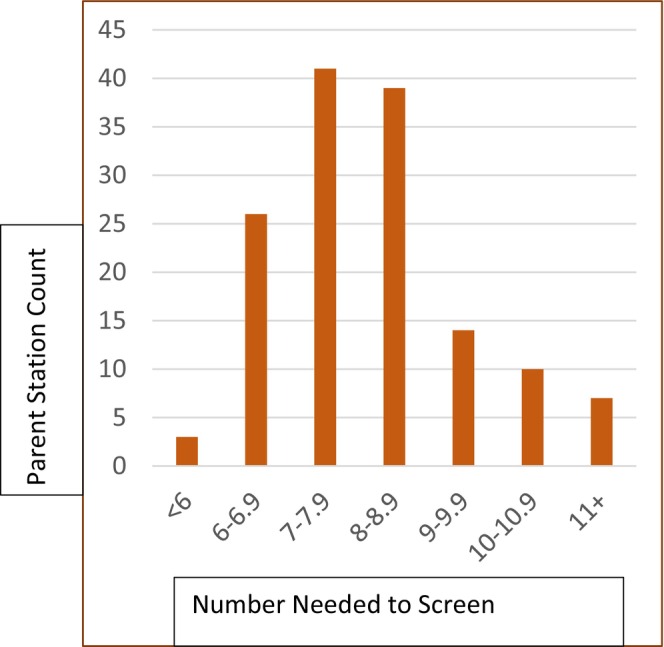
Distribution of Number Needed to Screen to identify one LTI using PLI across the 139 VA Medical Center (parent facilities). Most parent facilities needed to screen between 7 and 8.9 Veterans to find one Veteran who will need LTI. About 11 parent facilities needed to screen more than 10, but 34 needed to screen less than 7.

The NNS range was from under 6 in one parent facility to greater than 11 in seven parent facilities, with 70 of the 139 parent facilities having NNS less than 8 (Figure 3). Ten‐fold cross validation showed narrow ranges for both NNS (8.03–8.09) and Sensitivity (0.364–0.368) (Table S3).

Table 1 also presents the average marginal effect on LTI among Veterans in the ER and CR strata of each of the PLI model covariates (based on model 8). Most covariates had positive average marginal effects in both strata, but prior hospitalization had a positive average marginal effect in the ER strata and a negative average marginal effect in the CR strata. Prior SNF use and prior LTI were significantly associated with future LTI. Heart failure (HF), obesity, sepsis, and male sex were not statistically significantly associated with LTI in the ER stratum. The logistic model parameter estimates are found in Table S2a. Model sensitivity was better in the ER stratum (ER 0.59; CR 0.27), while discrimination was better in the CR stratum (c‐statistic: ER, 0.77; CR, 0.89), in part because of the lower LTI prevalence in the CR stratum. Pseudo r^2^ was greater in the CR strata (0.202) than in the ER strata (0.108).

### 3.3 Classifying the Veteran Population by PLI Risk Tiers

Table 2 presents the use of non‐institutional care and personal care services, and average total VA cost in FY2018 as well as rates of LTI and death in FY2018–2019 for the PLI‐high risk, moderate risk, and low risk tiers. The PLI‐high risk tier included 3.8% of VA users with 12.3% entering LTI, accounting for 41% of all veterans entering LTI over the next 2 years. The 2‐year mortality was 35% compared to 19% in the PLI‐MR tier. In FY2018, 42% of the PLI‐high risk tier received non‐institutional care services, including 22% who received personal care services. The PLI‐high risk tier, though based on LTI risk, identified 11.3% (80,552/710,823) of all veterans who were hospitalized in 2018–2019 with a positive predictive value of 0.44.

**TABLE 2:** Prior utilization, cost and future long‐term institutionalization and death.

	Predicted long‐term institutionalization (PLI)		
	High risk	Moderate risk	Low risk
*N*	208,061	479,129	4,757,163
	(3.8%)	(8.8%)	(87.4%)
Primary outcome			
LTI in 2 years, *N* (%)^a^	25,625	18,934	17,316
	(12.3%)	(4.0%)	(0.36%)
Secondary outcomes			
Death in 2 years, *N* (%)	72,721	91,229	145,000
	(35.0%)	(19.0%)	(3.1%)
VA costs per Veteran, mean (SD)	$39,178	$23,845	$7875
GEC NIC use, *N* (%)^b^	86,399	85,498	104,778
	(41.5%)	(17.8%)	(2.2%)
GEC PCS use, *N* (%)^c^	45,313	31,973	26,388
	(21.8%)	(6.7%)	(0.6%)

*Note:* Outcomes across three tiers of LTI risk (Model 8) for 5,444,354 veterans using VA in FY2017 and alive on the last day of FY2017 excluding those with LTI, in hospital, or nursing home on the last day of FY2017, and those who did not have any face‐to‐face diagnoses in FY 2017.

^a^ Long‐term Institutionalization (LTI) defined as more than 100 consecutive days of nursing home (NH) care.

^b^ Geriatric extended care (GEC) Non‐institutional services (NIC).

^c^ Geriatric extended care (GEC) personal care services (PCS) as defined as homemaker or home‐health aide, respite, adult day health care, Veteran‐directed care, and program for all‐inclusive care of the elderly.

PLI‐high risk prevalence varied across regions and institutions. The percent of Veterans using VA care classified as PLI‐high risk exceeded 4% in 69 of the 139 medical centers, and 6% in 26 medical centers (Figure S3).

### 3.4 PLI‐Production Model (Model 9)

We identified 206,786 Veterans as PLI‐high risk, capturing 24,176 Veterans experiencing LTI (39.1% sensitivity) with a NNS of 8.5. With a threshold of 6% LTI risk for the ER stratum and 7% LTI risk for the CR stratum, the PLI‐high risk tier was composed of two‐thirds from the ER stratum and one‐third from the CR stratum (Figure 1). Specificity was similar (96.5% vs. 96.6%), although the positive predictive value for death and LTI was higher at 0.5 (Table S1).

C‐statistics and pseudo r^2^ for PLI‐production were comparable to the PLI model for both ER and CR segments (Table S4). In both strata, the PLI‐production average marginal effects for most of the variables were outside the 95% confidence intervals of the PLI model (Table S4). However, the direction of the estimate was reversed only for Morbid Obesity in the ER strata of PLI‐production.

### 3.5 Sensitivity Analyses

We re‐estimated Model 8 as a probit model, with probit parameter estimates presented in Table S2b, and compared to the logistic model in Table S2c. Heart failure, while not statistically related to LTI in the CR stratum when race was age‐centered (Model 8), was marginally significant in the probit CR stratum (*p* = 0.049). Pseudo r^2^ was marginally greater in the probit than logistic models for both CR strata (0.21 probit, 0.202 logistic) and ER strata (0.125 probit, 0.108 logistic). Probit Model 8 had inferior performance, with a sensitivity of 36.6%, specificity 97.5%, with 193,681 Veterans identified in the high risk tier, representing 22,929 LTI, for a NNS of 8.4.

When applied to sequential out of sample cohorts from FY 2013–2017, the distribution of Veterans in risk tiers remained stable, including the PLI‐high risk tier and the share entering LTI each year (Figure S4). Specifically, for the FY2015 cohort (outcomes measures FY2016‐7), PLI had a similar sensitivity (41.4% vs. 41.1%), specificity (96.2% vs. 96.6%) and NNS (8.4 vs. 8.1) (Table S5).

## 4 Discussion

Despite the limits of administrative data for predicting LTI, PLI identified 41% of Veterans who experienced LTI within 2 years, with 8 Veterans needed to screen to identify one new LTI user, employing a mix of diagnostic, demographic, utilization, and risk measures constructed with combined VA and CMS data on two risk strata. Using this measure to proactively guide comprehensive geriatric assessment and targeting services may allow VA to improve its current deployment of Home & Community Based Services (HCBS) to those at highest risk of LTI [2, 5, 6, 30, 31]. Having both high discrimination and being well calibrated, these LTI risks can be used for either prediction (as intended in RECAP targeting) or adjustment for LTI risk in program evaluations. The use of age normalization improved model performance (effectively stratifying only age by race), better accounting for the differential age distribution of LTI entry among White and Black Veterans, without imposing current racial patterns of services for the other covariates.

Our analyses showed that less than 23% of high‐risk Veterans entering nursing homes long‐term had received personal care services in the year before entry, consistent with prior reports [2]. Given that nearly 60% of new LTI comes from non‐high‐risk tiers, expansion of HCBS, rather than reallocation, may be necessary to limit the projected rise in long‐term nursing home use.

The increase in the share of identified Veterans entering LTI resulted from the incorporation of Traditional Medicare and MDS data, a stratified risk model, and racially centered age. Incorporation of Medicare data both broadened Veterans' diagnoses by 43% and identified types of care (e.g., SNF use) that were predictive of future LTI and ER status (post‐acute care, one of the criteria for IAH‐Q). Medicare data were similarly crucial for the split stratum estimation (two‐thirds of whom were IAH‐Q), which improved sensitivity by 31%. More timely access to Medicare data is thus essential.

In the past, substantial delays in obtaining CMS data precluded incorporation of Medicare data into VA risk models [32] despite the substantial dual use of VA and Medicare services [33]. Recent process changes have reduced those lags to 6–9 months. We accounted for the missing Medicare data from 6 months prior to index by extending the look‐back period an additional 12 months and creating categorical flags for conditions present in the prior 13–24 months that may impact risk at the target date. Most important were LTI use, SNF use, and placing the Veteran in the ER strata by improving identification of IAH‐Q status. While resulting in similar model performance, the individuals identified by the two approaches (most recent 6 months of data—available in the complete data model—versus an additional 12 prior months of data for the production model) identify different populations of Veterans, with about 78% of PLI‐high risk tier Veterans identified in both models.

As the purpose of the model was to identify a high‐risk population to screen for specific deficits and needs, the target number needed to screen was set at 8 by Geriatric and Extended Care leadership, based in part on the prior *Choose Home* pilot [2]. Thresholds were chosen so the greatest number of LTI Veterans were identified (maximizing sensitivity), subject to an overall average NNS of 8. The resulting thresholds of 6% for the ER stratum and 7% for the CR stratum had similar marginal NNS (12:1 at the 6% margin for ER and 11:1 at the 7% margin for CR). This constraint limited the model's operating sensitivity to 41%; sensitivity could be increased at the cost of screening more Veterans. Other models predicting LTI among Veterans have shown the importance of survey data to collect functional and family support information, with higher sensitivity, but set at levels with a NNS of 17 [9], with overall performance (c statistic) comparable to PLI.

The model has several limitations. First, being administrative data based, it lacks key measures of family supports, social determinants, physical and cognitive function, and challenging behaviors, all factors that have been shown to be important in the decision of LTI [7, 8, 9]. Second, while incorporating LTI use for all Veteran Medicare beneficiaries, the available Medicare data for predictors in this study excluded those for the 25% of Medicare enrolled Veterans using Medicare Advantage in 2017, increasing by 2022 to 40% of Veterans enrolled in Medicare [34]. As the completeness of Medicare Advantage records has improved since 2017, they should be incorporated into future iterations of the PLI. We also lacked Medicaid data, although PLI is agnostic to HCBS measures of which Medicaid is a major payer, and Medicaid‐paid NH use would be captured by MDS, as it is only paid for in CMS‐certified nursing homes, and incremental diagnostic information from Medicaid beyond VA and Medicare sources for the 9.2% of Medicaid‐enrolled VA users is likely minimal. Third, PLI captures behavior around NH use before the SARS‐CoV‐2 pandemic. There have been reports of lower LTI use post‐pandemic [35, 36]. By September 2025, we will have sufficient data to recalibrate the model in the post‐pandemic period and explore modifications to adjust for post‐pandemic changes in behavior.

## 5 Conclusions

The PLI score is useful for identifying LTI risk in a population, using secondary data sources to identify a high‐risk group for clinical assessment. The same group also has high risks of death, hospitalization, and cost—outcomes for other targeted clinical programs. With attention to data lags, clarification of CMS‐VHA data arrangements can usefully allow incorporation of Medicare data into analytic tools to improve Veteran care.

## Conflicts of Interest

The authors declare no conflicts of interest, except that all are (or were at the time of this work) employees of the Department of Veterans Affairs.

## Supporting information


**Data S1.** Supporting Information.

## Data Availability

The data that support the findings of this study are available on request from the corresponding author. The data are not publicly available due to privacy or ethical restrictions.
