# Supplementary material for: Predicting Risk of Long‐Term Institutionalization Among Community Dwelling Veterans Before the COVID‐19 Pandemic
Source: Health Serv Res. 2025 Jul 26;61(1):e70016. doi: 10.1111/1475-6773.70016 (PMC12857474; doi:10.1111/1475-6773.70016)
Supplement: Supplementary file 1 — Data S1. Supporting Information. [file HESR-61-e70016-s001.docx]

**Supplementary Materials**

**eFigure 1 :Population flow**

**eTable 1: Model development**

**eTable 2: 2a Full Model—Logistic**

**2b Full Model—probit**

**2c AMEs for logistic and probit comparison**

**eTable 3: Cross-validation results**

**eTable 4: PLI-P/PLI covariate comparison**

**eFigure 2: Age at death and LTI by race**

**eFigure 3: High risk Veteran distribution by facility**

**eFigure 4: Risk tier stability 2013-2017**

**eTable 5 : FY2017 PLI applied to FY2015 cohort**

**eFigure 5: Calibration comparison Age and Age-Centered**

**eFigure 6a: Black and White Veteran calibration differences Logistic model**

**6b: Black and White Veteran calibration differences Logistic and Probit models**

**S2: TRIPOD checklist**

**eFigure 1. Selection of study population from all Veteran users.**

**eTable 1a Model Development**

The PLI model was developed in stages with each stage adding data sources, variables and stratification, in order to monitor the importance of each stage. We started from a logistic model predicting a single outcome identifying LTI or death as presented in a previous study^2^ using only VA data (to compute both covariates and outcomes) (Model 0). Modelling stages were assessed were as follows:

Model 1: Added Medicare enrollment and claims data and MDS assessments from community nursing homes to re-compute a more precise LTI outcome.

Model 2: Stratified Risk Models. Estimated two separate risk models for strata of Elevated Risk (ER) and Common Risk (CR). ER stratum was determined by presence of any Independence-at-Home qualification (IAH-Q) or High Needs High Risk criteria.

Model 3: Additional covariates and ICD10 diagnoses. We added utilization covariates (prior LTI, prior SNF) and an expanded ICD-10 code list for diagnoses using only VA data.

Model 4: JEN Frailty Index as covariate. We calculated JFI based on VA diagnoses and included as covariate in the model.

Model 5: Included Medicare data to covariates. We updated JFI and comorbidities to include Medicare data.

Model 6: Parent station fixed effects. The VA has 139 parent facilities, which serve as administrative and clinical hubs with hospital and specialty services. We assigned each patient to a home facility. By adding home facility fixed effects, or indicators of 139 parent facilities, we can control for local organizational factors, culture, and health practices, such as availability of non-VA community supports and services.

Model 7: Split Thresholds for Elevated Risk (ER) and Common Risk (CR) Strata. We identified different LTI risk thresholds for the ER and CR PLI models to improve sensitivity while maintaining number needed to screen (NNS) is no more than 8.

Model 8: FINAL PLI MODEL (PLI) Centered age at racial averages to correct for racial differences in LTI risk. We updated the age measure to correct the negative bias (lower rate of high-risk tier) on Black Veteran LTI risk.

Model 9: PLI MODEL FOR PRODUCTION (PLI-P) Updated variables and data with 2-year look back in Medicare data.

**eTable 1.** Performance of LTI models among Veteran patients comparing simple logistic regression model using only VA data to addition of Medicare data, stratification to elevated and low risk models, expanded covariate list, addition of other covariates, addition of frailty measure, two years lookback, VA facility fixed effects and different thresholds for elevated and low risk models. Models were run on 5,466,598 Veteran with 62,056 entering LTI and 371,607 dying in fiscal years 2018-2019. The elevated stratum included 456,921 Veterans with 24,050 LTI and 93,702 death, and the low-risk stratum included 5,009,677 Veterans with 37,852 LTI and 215,318 deaths in FY2018-9.

| **Model Steps** | | **PLI High Risk Threshold**  **(ER / CR )** | **PLI High Risk (N)** | **Observed LTI (N) in HR Tier** | **Sensitivity** | **Specificity** | **Increased**  **Sensitivity compared to Baseline** | **PPV** | **NNS** |
| --- | --- | --- | --- | --- | --- | --- | --- | --- | --- |
| **Model 0.** VA data only for covariates | | 0.5 (PPV for LTI or death) | 66,913 | 3,986 | 6.4% |  |  | 0.49 | 16.8 |
| **Model 1.** Updated Outcome to include Medicare + MDS data for LTI | | .07 | 118,061 | 14,340 | 23.1% | 98.1% | Baseline | 0.50 | 8.2 |
| **Model 2.** Stratified PLI Models for elevated risk (ER) and common risk (CR) | | .07 / .07 | 157,158 | 18,761 | 30.2% | 97.4% | 31% | 0.47 | 8.4 |
| **Model 3.** Additional covariates and ICD10 diagnoses | | .07 / .07 | 157,818 | 18,719 | 30.2% | 97.4% | 31% | 0.46 | 8.4 |
| **Model 4:** JEN Frailty Index as covariate | | .07 / .07 | 158,153 | 18,788 | 30.3% | 97.4% | 31% | 0.47 | 8.4 |
| **Model 5**: Include Medicare data to covariates | | .07 / .07 | 161,955 | 20,615 | 33.2% | 97.4% | 44% | 0.48 | 8.1 |
| **Model 6:** Parent Station fixed effects | | .07 / .07 | 162,998 | 21,106 | 34.1% | 97.4% | 47% | 0.47 | 7.7 |
| **Model 7**. Split threshold for ER and CR strata **(PLI split threshold)** | | .06 / .07 | 183,386 | 22,703 | 36.6% | 97.0% | 58% | 0.46 | 8.1 |
| **Model 8.** Normalized Age for racial differences in LTI risk **(PLI)** | | .06 / .07 | 208,061 | 25,626 | 41.1% | 96.6% | 78% | 0.38 | 8.1 |
| **Model 9.** Normalized Age PLI Production **(PLI-P)** | | .06 / .07 | 206,786 | 24,176 | 39.1% | 96.5% | 69% | 0.50 | 8.5 |
|  | Elevated Risk (ER); Common Risk (CR)  Minimum Data Set (MDS); JEN Frailty index (JFI);  Predicted Long-term Institutionalization (PLI); Long-term Institutionalization (LTI);  Positive Predictive Value (PPV); Number Needed to Screen (NNS); | | | | | | | | |

**eTable 2a. Final PLI Model estimates across Elevated Risk (ER) and Common Risk (CR) strata.** This is model 8 (logistic) from complete data and age-normalized measures. ER and CR strata were determined by having or not meeting either Independence-at-Home qualification (IAH-Q) or High Needs High Risk criteria. CHF, while not significant in the age-normalized model 8, was significant in the CR strata in the standard age model (model 7).

ER: c-statistic = 0.782, pseudo r^2^ =0.108; CR: c-statistic = 0.878, pseudo r^2^ = 0.202

|  | **Elevated Risk** | |  | **Common Risk** | |  |
| --- | --- | --- | --- | --- | --- | --- |
|  | **Coefficient** | **Std. err.** | **P>z** | **Coefficient** | **Std. err.** | **P>z** |
| Center Age_ Race | -4.683 |  | <.0001 | -.073 |  | <.0001 |
| Male | .045 | .000 | 0.2310 | .131 | .000 | <.0001 |
| Married | -.347 | .001 | <.0001 | -.437 | .000 | <.0001 |
| Rural | -.248 | .001 | <.0001 | -.377 | .000 | <.0001 |
| Priority 1 | -.061 | .001 | <.0001 | -.129 | .000 | <.0001 |
| Homeless | .362 | .000 | <.0001 | .602 | .000 | <.0001 |
| JFI (VA and MC dx) | .084 | .002 | <.0001 | .066 | .001 | <.0001 |
| CAN prob of 1Yr event | .339 | .000 | <.0001 | 1.548 | .000 | <.0001 |
| Missing CAN | .765 | .000 | <.0001 | .601 | .000 | <.0001 |
| Acute hospital stay | .185 | .001 | <.0001 | -.192 | .000 | <.0001 |
| Prior LTI | .780 | .000 | <.0001 | .985 | .000 | <.0001 |
| Prior SNF | .308 | .001 | <.0001 | .795 | .000 | <.0001 |
| VA total cost (x10,000) | .018 | .008 | <.0001 | .036 | .001 | <.0001 |
| Amputation | .257 | .000 | <.0001 | .651 | .000 | <.0001 |
| Cancer | -.275 | .001 | <.0001 | -.265 | .000 | <.0001 |
| CHF | -.010 | .001 | 0.4721 | .007 | .000 | 0.6710 |
| Dementia | .850 | .001 | <.0001 | 1.363 | .000 | <.0001 |
| Diabetes | .142 | .001 | <.0001 | .197 | .000 | <.0001 |
| Fracture | .294 | .000 | <.0001 | .334 | .000 | <.0001 |
| Head Injury | -.171 | .000 | <.0001 | -.363 | .000 | <.0001 |
| Malnutrition | .164 | .000 | <.0001 | .378 | .000 | <.0001 |
| Multiple Sclerosis | .431 | .000 | <.0001 | .953 | .000 | <.0001 |
| Obesity | -.015 | .001 | 0.3562 | -.133 | .000 | <.0001 |
| Parkinson's | .456 | .000 | <.0001 | .945 | .000 | <.0001 |
| Pressure Ulcer | .404 | .000 | <.0001 | .578 | .000 | <.0001 |
| Schizophrenia | .599 | .000 | <.0001 | .980 | .000 | <.0001 |
| Spinal Cord Injury | .157 | .000 | <.0001 | .573 | .000 | <.0001 |
| Seizure | .175 | .000 | <.0001 | .381 | .000 | <.0001 |
| Sepsis | -.014 | .000 | 0.4402 | .206 | .000 | <.0001 |
| Stroke | .387 | .000 | <.0001 | .565 | .000 | <.0001 |
| Substance use disorder | .083 | .001 | <.0001 | .145 | .000 | <.0001 |

**eTable 2b Probit estimates of PLI Model 8 Elevated Risk (ER) and Common Risk (CR) strata;**

|  | **Elevated Risk** | |  | **Common Risk** | |  |
| --- | --- | --- | --- | --- | --- | --- |
|  | **Coefficient** | **Std. err.** | **P>z** | **Coefficient** | **Std. err.** | **P>z** |
| CenterAge_Race | -0.02 | 0.00 | 0.00 | -0.028 | 0.000 | 0.000 |
| Married | -0.17 | 0.01 | 0.00 | -0.182 | 0.005 | 0.000 |
| Male | 0.02 | 0.02 | 0.36 | 0.039 | 0.012 | 0.001 |
| Priority 1 | -0.03 | 0.01 | 0.00 | -0.060 | 0.005 | 0.000 |
| CAN 1Y event prob | 0.18 | 0.02 | 0.00 | 0.803 | 0.021 | 0.000 |
| Missing CAN | 0.39 | 0.02 | 0.00 | 0.266 | 0.011 | 0.000 |
| JFI (VA and MC dx) | 0.04 | 0.00 | 0.00 | 0.027 | 0.001 | 0.000 |
| Prior SNF | 0.16 | 0.01 | 0.00 | 0.388 | 0.014 | 0.000 |
| Prior LTI | 0.43 | 0.02 | 0.00 | 0.498 | 0.024 | 0.000 |
| Acute Hosp Stay | 0.08 | 0.01 | 0.00 | -0.101 | 0.009 | 0.000 |
| VA total cost (x$10,000 | 0.01 | 0.00 | 0.00 | 0.016 | 0.001 | 0.000 |
| Rural | -0.12 | 0.01 | 0.00 | -0.155 | 0.006 | 0.000 |
| Homeless | 0.17 | 0.02 | 0.00 | 0.221 | 0.013 | 0.000 |
| Dementia | 0.43 | 0.01 | 0.00 | 0.637 | 0.007 | 0.000 |
| Parkinson's | 0.24 | 0.01 | 0.00 | 0.454 | 0.011 | 0.000 |
| Schizophrenia | 0.31 | 0.01 | 0.00 | 0.407 | 0.013 | 0.000 |
| Substance use Disorder | 0.04 | 0.01 | 0.00 | 0.040 | 0.009 | 0.000 |
| Cancer | -0.13 | 0.01 | 0.00 | -0.116 | 0.007 | 0.000 |
| CHF | 0.00 | 0.01 | 0.93 | 0.015 | 0.007 | 0.049 |
| Diabetes | 0.07 | 0.01 | 0.00 | 0.074 | 0.005 | 0.000 |
| Fracture | 0.16 | 0.01 | 0.00 | 0.166 | 0.026 | 0.000 |
| Head Injury | -0.08 | 0.02 | 0.00 | -0.135 | 0.019 | 0.000 |
| Amputation | 0.13 | 0.02 | 0.00 | 0.266 | 0.021 | 0.000 |
| Malnutrition | 0.09 | 0.01 | 0.00 | 0.215 | 0.021 | 0.000 |
| Multiple Sclerosis | 0.24 | 0.03 | 0.00 | 0.410 | 0.030 | 0.000 |
| Obesity | -0.01 | 0.01 | 0.22 | -0.059 | 0.007 | 0.000 |
| Pressure Ulcer | 0.21 | 0.01 | 0.00 | 0.295 | 0.011 | 0.000 |
| Seizure | 0.09 | 0.01 | 0.00 | 0.177 | 0.014 | 0.000 |
| Sepsis | 0.00 | 0.01 | 0.88 | 0.106 | 0.023 | 0.000 |
| Spinal Cord Injury | 0.09 | 0.01 | 0.00 | 0.259 | 0.021 | 0.000 |
| Stroke | 0.20 | 0.01 | 0.00 | 0.272 | 0.009 | 0.000 |

**eTable** 2c **Comparison of average marginal effects (AME) of Probit and Logistic Model 8** Probit AMEs outside the 95% logistic CIs (brown for higher, green for lower )

| **Common Risk** |  | |  | |  | |  | |  | |
| --- | --- | --- | --- | --- | --- | --- | --- | --- | --- | --- |
|  | | **Logistic** | | | | | | **Probit** | |  |
|  | | **AME** | | **[95% Confidence Interval]** | | | | **AME** | |  |
| CenterAge_Race | | -0.00049 | | -0.0005 | | -0.0004 | | -0.00047 | |  |
| Married | | -0.00294 | | -0.0030 | | -0.0027 | | -0.00300 | |  |
| Male | | 0.00088 | | 0.00046 | | 0.00130 | | 0.00065 | |  |
| Priority 1 | | -0.00087 | | -0.0010 | | -0.0007 | | -0.00098 | |  |
| CAN 1Y event prob | | 0.01040 | | 0.00979 | | 0.01102 | | 0.01321 | |  |
| Missing CAN | | 0.00404 | | 0.00366 | | 0.00441 | | 0.00438 | |  |
| JFI (VA and MC dx) | | 0.00044 | | 0.00040 | | 0.00048 | | 0.00045 | |  |
| Prior SNF | | 0.00534 | | 0.00495 | | 0.00573 | | 0.00637 | |  |
| Prior LTI | | 0.00662 | | 0.00595 | | 0.00729 | | 0.00819 | |  |
| Acute Hosp Stay | | -0.00129 | | -0.0015 | | -0.0010 | | -0.00165 | |  |
| VA total cost (x$10,000) | | 0.00024 | | 0.00022 | | 0.00027 | | 0.00026 | |  |
| Rural | | -0.00253 | | -0.0027 | | -0.0023 | | -0.00255 | |  |
| Homeless | | 0.00404 | | 0.00360 | | 0.00449 | | 0.00364 | |  |
| Dementia | | 0.00916 | | 0.00895 | | 0.00937 | | 0.01047 | |  |
| Parkinson's | | 0.00635 | | 0.00603 | | 0.00668 | | 0.00747 | |  |
| Schizophrenia | | 0.00659 | | 0.00618 | | 0.00699 | | 0.00670 | |  |
| Substance use Disorder | | 0.00097 | | 0.00068 | | 0.00127 | | 0.00066 | |  |
| Cancer | | -0.00178 | | -0.0020 | | -0.0015 | | -0.00191 | |  |
| CHF | | 0.00005 | | -0.0001 | | 0.00028 | | 0.00024 | |  |
| Diabetes | | 0.00132 | | 0.00117 | | 0.00148 | | 0.00121 | |  |
| Fracture | | 0.00225 | | 0.00149 | | 0.00301 | | 0.00272 | |  |
| Head Injury | | -0.00244 | | -0.0031 | | -0.0017 | | -0.00222 | |  |
| Amputation | | 0.00438 | | 0.00374 | | 0.00501 | | 0.00438 | |  |
| Malnutrition | | 0.00254 | | 0.00193 | | 0.00315 | | 0.00354 | |  |
| Multiple Sclerosis | | 0.00641 | | 0.00546 | | 0.00735 | | 0.00674 | |  |
| Obesity | | -0.00089 | | -0.0011 | | -0.0006 | | -0.00097 | |  |
| Pressure Ulcer | | 0.00389 | | 0.00355 | | 0.00422 | | 0.00485 | |  |
| Seizure | | 0.00256 | | 0.00214 | | 0.00298 | | 0.00292 | |  |
| Sepsis | | 0.00139 | | 0.00069 | | 0.00208 | | 0.00174 | |  |
| Spinal Cord Injury | | 0.00385 | | 0.00321 | | 0.00449 | | 0.00425 | |  |
| Stroke | | 0.00380 | | 0.00352 | | 0.00408 | | 0.00448 | |  |

**Elevated Risk**

|  | **Logistic** | | | **Probit** |
| --- | --- | --- | --- | --- |
|  | **AME** | **[95% Confidence Interval]** | | **AME** |
| CenterAge_Race | -0.00144 | -0.00151 | -0.00137 | -0.00146 |
| Married | -0.01579 | -0.01702 | -0.01455 | -0.0159 |
| Male | 0.00204 | -0.00129 | 0.00536 | 0.00149 |
| Priority 1 | -0.00279 | -0.00410 | -0.00148 | -0.0031 |
| CAN 1Y event prob | 0.01540 | 0.01239 | 0.01841 | 0.01701 |
| Missing CAN | 0.03476 | 0.03202 | 0.03751 | 0.03593 |
| JFI (VA and MC dx) | 0.00383 | 0.00335 | 0.00431 | 0.00382 |
| Prior SNF | 0.01399 | 0.01254 | 0.01544 | 0.01531 |
| Prior LTI | 0.03543 | 0.03171 | 0.03915 | 0.04016 |
| Acute Hosp Stay | 0.00841 | 0.00689 | 0.00993 | 0.00713 |
| VA total cost (x$10,000) | 0.00080 | 0.00071 | 0.00089 | 0.00084 |
| Rural | -0.01126 | -0.01290 | -0.00961 | -0.0113 |
| Homeless | 0.01646 | 0.01367 | 0.01924 | 0.01599 |
| Dementia | 0.03864 | 0.03720 | 0.04008 | 0.04040 |
| Parkinson's | 0.02070 | 0.01853 | 0.02288 | 0.02274 |
| Schizophrenia | 0.02720 | 0.02460 | 0.02981 | 0.02851 |
| Substance use Disorder | 0.00379 | 0.00206 | 0.00551 | 0.00350 |
| Cancer | -0.01248 | -0.01395 | -0.01102 | -0.0125 |
| CHF | -0.00047 | -0.00176 | 0.00081 | 0.00006 |
| Diabetes | 0.00644 | 0.00519 | 0.00769 | 0.00650 |
| Fracture | 0.01334 | 0.01102 | 0.01565 | 0.01462 |
| Head Injury | -0.00779 | -0.01068 | -0.00490 | -0.0076 |
| Amputation | 0.01167 | 0.00894 | 0.01439 | 0.01184 |
| Malnutrition | 0.00746 | 0.00557 | 0.00935 | 0.00871 |
| Multiple Sclerosis | 0.01957 | 0.01387 | 0.02528 | 0.02191 |
| Obesity | -0.00069 | -0.00215 | 0.00078 | -0.0008 |
| Pressure Ulcer | 0.01837 | 0.01679 | 0.01995 | 0.02000 |
| Seizure | 0.00796 | 0.00594 | 0.00998 | 0.00863 |
| Sepsis | -0.00066 | -0.00233 | 0.00101 | -0.00013 |
| Spinal Cord Injury | 0.00712 | 0.00454 | 0.00971 | 0.00817 |
| Stroke | 0.01760 | 0.01614 | 0.01905 | 0.01884 |

**eTable 3:** Distribution of sensitivity, positive predicted value (ppv), and number needed to screen from 10-fold cross validation for PLI at both constant (Model 6) and split (Model 7) thresholds for ER and CR strata.

| **Variable** | **Mean** | **Std Dev** | **Median** | **Min** | **Max** |
| --- | --- | --- | --- | --- | --- |
| **Model 6. Thresholds ER=.07; CR=.07** |  |  |  |  |  |
| **Sensitivity** | **0.34** | **.002** | **.34** | **.34** | **.34** |
| **PPV** | **.47** | **.0008** | **.471** | **.47** | **.47** |
| **NNS** | **7.71** | **.031** | **7.72** | **7.66** | **7.73** |
| **Model 7. Thresholds**  **ER=.06; CR=.07** |  |  |  |  |  |
| **Sensitivity** | **.36** | **.0015** | **.366** | **.36** | **.37** |
| **PPV** | **.45** | **.0006** | **.458** | **.458** | **.46** |
| **NNS** | **8.07** | **.03** | **8.07** | **8.03** | **8.09** |

**eTable 4**. **PLI Production (PLI-P) Models for the elevated (ER) and Common risk (CR) segments** Based on Medicare data that is lagging by 6 months, with PLI AME CIs as reference. This model uses a 2-year lookback period for its covariates (FY16-FY17). PLI-P AMEs that are greater than the PLI Upper Confidence Interval are highlighted in rose , PLI-P AMEs lower than the PLI Lower Confidence Interval are highlighted in green. The c-statistics for the PLI and PLI-P ER-models were .782 and .767, respectively, and .878 and .885 for the PLI and PLI-P CR models, respectively. For the CR model the pseudo R^2^ for PLI = .2022 and PLI-P= .1846, For the ER model, the pseudo R^2^ for PLI was .1079, and for PLI-P =.1022.

| **Elevated Risk** |  |  |  |  |
| --- | --- | --- | --- | --- |
|  | **AME** | **[95% confidence interval]** | | **PLI-P AME** |
| Center Age Race | -0.00144 | -0.00151 | -0.00137 | -0.0014132 |
| Married | -0.01579 | -0.01702 | -0.01455 | -0.0153184 |
| Male | 0.00204 | -0.00129 | 0.00536 | 0.002653 |
| Priority 1 | -0.00279 | -0.00410 | -0.00148 | -0.0038908 |
| CAN 1Y event prob | 0.01540 | 0.01239 | 0.01841 | 0.0338286 |
| Missing CAN | 0.03476 | 0.03202 | 0.03751 | 0.0446031 |
| JFI (VA and MC dx) | 0.00383 | 0.00335 | 0.00431 | 0.004859 |
| Prior SNF | 0.01399 | 0.01254 | 0.01544 | 0.0102408 |
| Prior LTI | 0.03543 | 0.03171 | 0.03915 | 0.0318068 |
| Acute Hosp Stay | 0.00841 | 0.00689 | 0.00993 | 0.0180195 |
| VA total cost (x$10,000 | 0.00080 | 0.00071 | 0.00089 | 0.0011874 |
| Rural | -0.01126 | -0.01290 | -0.00961 | -0.0130383 |
| Homeless | 0.01646 | 0.01367 | 0.01924 | 0.0257126 |
| Dementia | 0.03864 | 0.03720 | 0.04008 | 0.0348146 |
| Parkinson's | 0.02070 | 0.01853 | 0.02288 | 0.0189134 |
| Schizophrenia | 0.02720 | 0.02460 | 0.02981 | 0.024285 |
| Substance use Disorder | 0.00379 | 0.00206 | 0.00551 | 0.0084064 |
| Cancer | -0.01248 | -0.01395 | -0.01102 | -0.0149002 |
| CHF | -0.00047 | -0.00176 | 0.00081 | -0.0030262 |
| Diabetes | 0.00644 | 0.00519 | 0.00769 | 0.0073859 |
| Fracture | 0.01334 | 0.01102 | 0.01565 | 0.006406 |
| Head Injury | -0.00779 | -0.01068 | -0.00490 | -0.0070063 |
| Amputation | 0.01167 | 0.00894 | 0.01439 | 0.0087621 |
| Malnutrition | 0.00746 | 0.00557 | 0.00935 | 0.0014856 |
| Multiple Sclerosis | 0.01957 | 0.01387 | 0.02528 | 0.0208465 |
| Obesity | -0.00069 | -0.00215 | 0.00078 | 0.0016636 |
| Pressure Ulcer | 0.01837 | 0.01679 | 0.01995 | 0.0147888 |
| Seizure | 0.00796 | 0.00594 | 0.00998 | 0.0059659 |
| Sepsis | -0.00066 | -0.00233 | 0.00101 | -0.002153 |
| Spinal Cord Injury | 0.00712 | 0.00454 | 0.00971 | 0.003218 |
| Stroke | 0.01760 | 0.01614 | 0.01905 | 0.0137368 |
| **Common Risk** |  |  |  |  |
|  | **AME** | **[95% confidence**  **interval]** | | **PLI-P AME** |
| CenterAge_Race | -0.00049 | -0.00050 | -0.00048 | -0.0004943 |
| Married | -0.00294 | -0.00309 | -0.00279 | -0.0027075 |
| Male | 0.00088 | 0.00046 | 0.00130 | 0.0013249 |
| Priority 1 | -0.00087 | -0.00104 | -0.00070 | -0.0010886 |
| CAN 1Y event prob | 0.01040 | 0.00979 | 0.01102 | 0.0167581 |
| Missing CAN | 0.00404 | 0.00366 | 0.00441 | 0.004938 |
| JFI (VA and MC dx) | 0.00044 | 0.00040 | 0.00048 | 0.0006563 |
| Prior SNF | 0.00534 | 0.00495 | 0.00573 | 0.0058927 |
| Prior LTI | 0.00662 | 0.00595 | 0.00729 | 0.0065201 |
| Acute Hosp Stay | -0.00129 | -0.00159 | -0.00100 | 0.0024311 |
| VA total cost (x$10,000 | 0.00024 | 0.00022 | 0.00027 | 0.0003527 |
| Rural | -0.00253 | -0.00272 | -0.00234 | -0.0028626 |
| Homeless | 0.00404 | 0.00360 | 0.00449 | 0.0067253 |
| Dementia | 0.00916 | 0.00895 | 0.00937 | 0.0076482 |
| Parkinson's | 0.00635 | 0.00603 | 0.00668 | 0.0055743 |
| Schizophrenia | 0.00659 | 0.00618 | 0.00699 | 0.0060187 |
| Substance use Disorder | 0.00097 | 0.00068 | 0.00127 | 0.0022501 |
| Cancer | -0.00178 | -0.00201 | -0.00156 | -0.0028971 |
| CHF | 0.00005 | -0.00018 | 0.00028 | -0.0005974 |
| Diabetes | 0.00132 | 0.00117 | 0.00148 | 0.0005052 |
| Fracture | 0.00225 | 0.00149 | 0.00301 | 0.0014422 |
| Head Injury | -0.00244 | -0.00310 | -0.00178 | -0.001532 |
| Amputation | 0.00438 | 0.00374 | 0.00501 | 0.0039431 |
| Malnutrition | 0.00254 | 0.00193 | 0.00315 | 0.001149 |
| Multiple Sclerosis | 0.00641 | 0.00546 | 0.00735 | 0.0062553 |
| Obesity | -0.00089 | -0.00112 | -0.00066 | -0.0009279 |
| Pressure Ulcer | 0.00389 | 0.00355 | 0.00422 | 0.0025661 |
| Seizure | 0.00256 | 0.00214 | 0.00298 | 0.001457 |
| Sepsis | 0.00139 | 0.00069 | 0.00208 | 0.0003596 |
| Spinal Cord Injury | 0.00385 | 0.00321 | 0.00449 | 0.0025815 |
| Stroke | 0.00380 | 0.00352 | 0.00408 | 0.0022817 |
|  |  |  |  |  |

**eFigure 2.** Age distributions of Age at Death and Age at Long-Term Institutionalization by Race (White, Black) among community dwelling Veterans, FY 2018-2019.


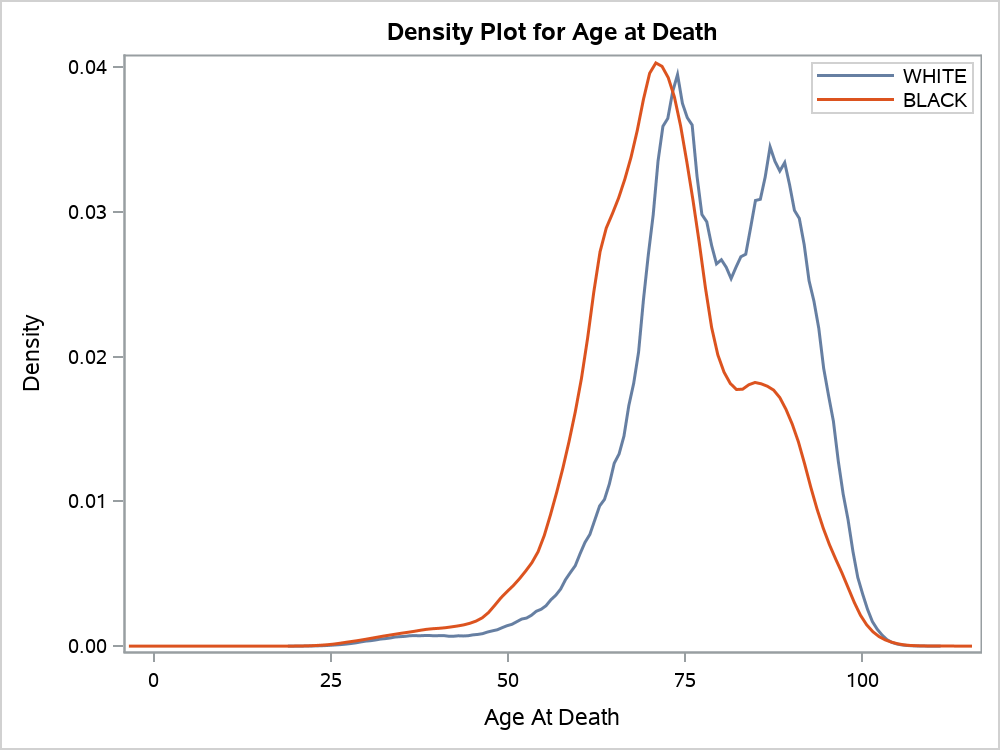


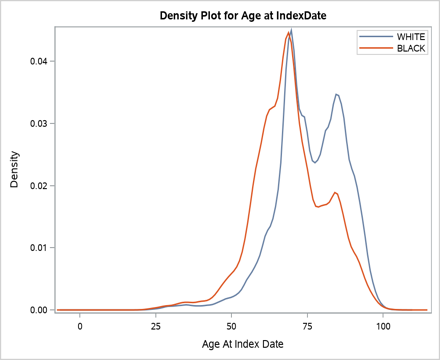


Age at LTI

Density Plot for Age at LTI

**eFigure 3**. Share of Veterans in 139 Parent facilities identified as High Risk in FY2018. (Model 8)

#
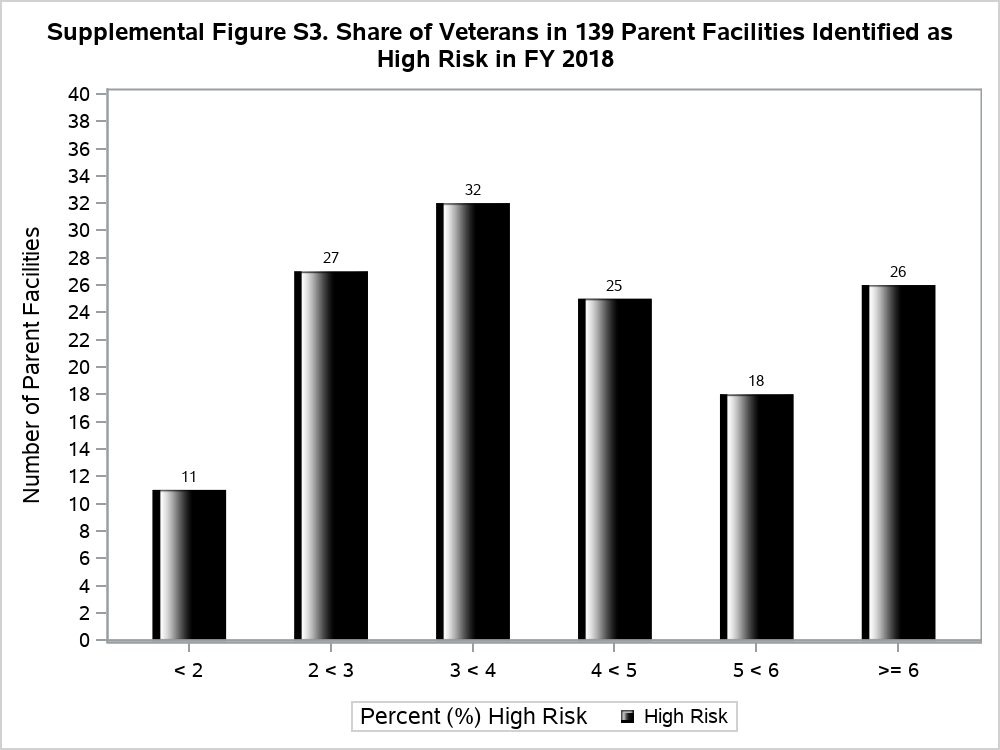


**eFigure 4** Application of PLI model 7 (calibrated on FY 2017) to VHA user cohorts using same selection criteria, assembled for FY 2013-2017. Detail shows consistent share of HR entering LTI each year.


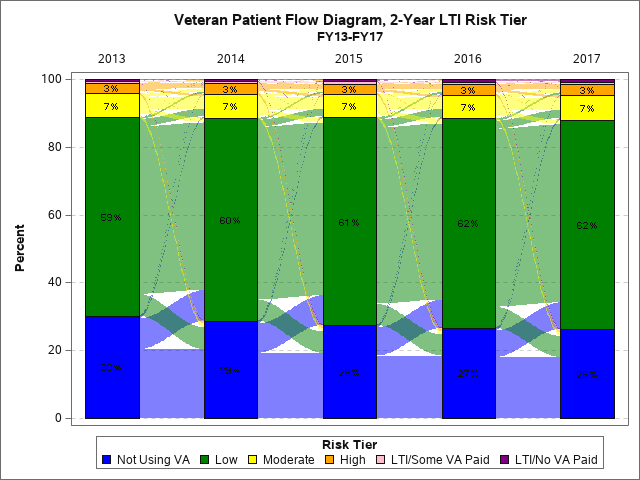


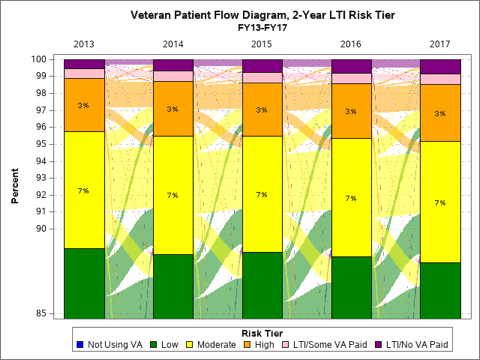


eTable 5 Out-of-sample prediction for FY2017 PLI (developed on FY2018-2019 cohort)

applied to similarly selected cohort predicting for FY2015 (outcomes FY2016-2017). N=5,743,564; High Risk Tier N=218,430 (3.8%)

| **Model Steps** | **PLI High Risk Threshold**  **(ER / CR )** | **PLI High Risk (N)** | **Observed LTI (N) in HR Tier** | **Sensitivity** | **Specificity** | **NNS** |
| --- | --- | --- | --- | --- | --- | --- |

| **Model 8.** Normalized Age for racial differences in LTI risk **(PLI)** | .06/.07 | 208,061 | 25,626 | 41.1% | 96.6% | 8.1 |
| --- | --- | --- | --- | --- | --- | --- |
| **Model 8 PLI (FY2017)**  **Applied to FY2015 cohort** | .06/.07 | 218,430 | 25,860 | 41.4% | 96.2% | 8.4 |

**eFigure 5.** Comparison of calibration of PLI with Age (insert) in Common (CR) and Elevated (ER) Risk groups to PLI with Race-centered (Normalized) Age in CR and ER.

**eFigure6a.**Calibration of PLI model 8 (normalized age) among White and Black Veterans.

Overall calibration is similar except for the 10^th^ decile of the Common Risk cohort among White Veterans, where the difference between Observed (.049) and Predicted (.041) risk is .008.

Elevated Risk


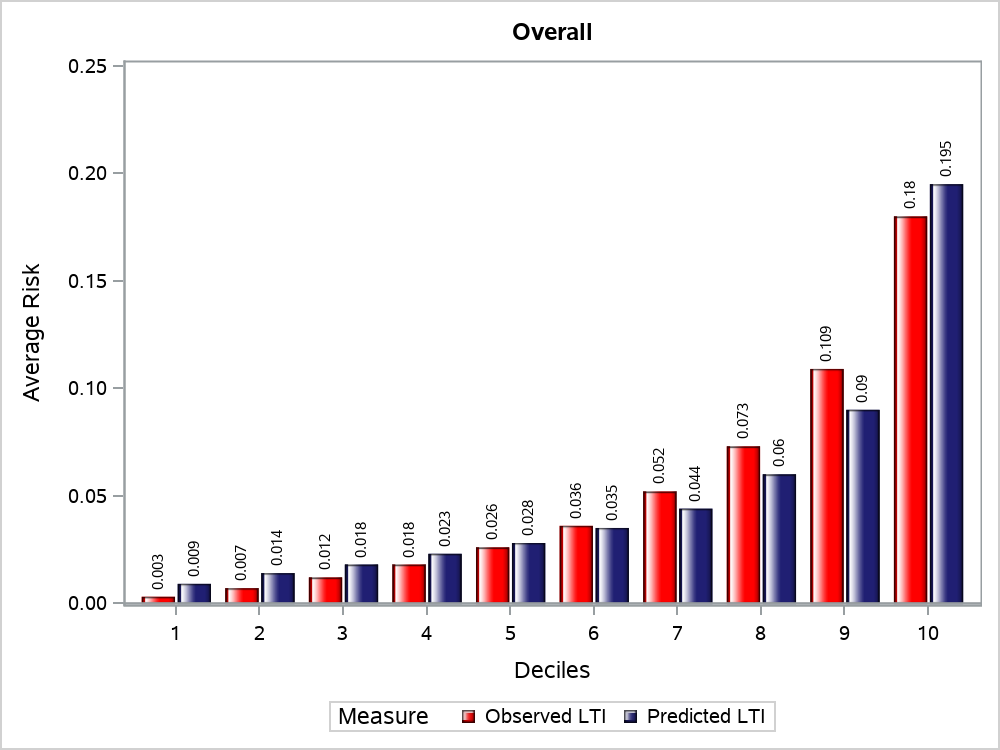

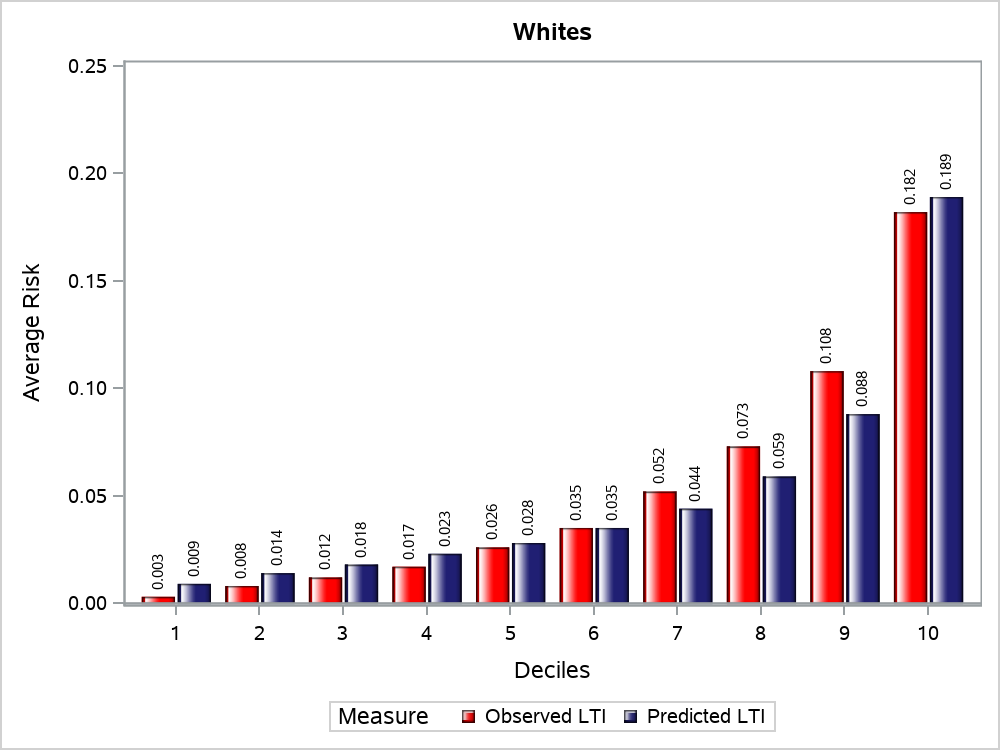

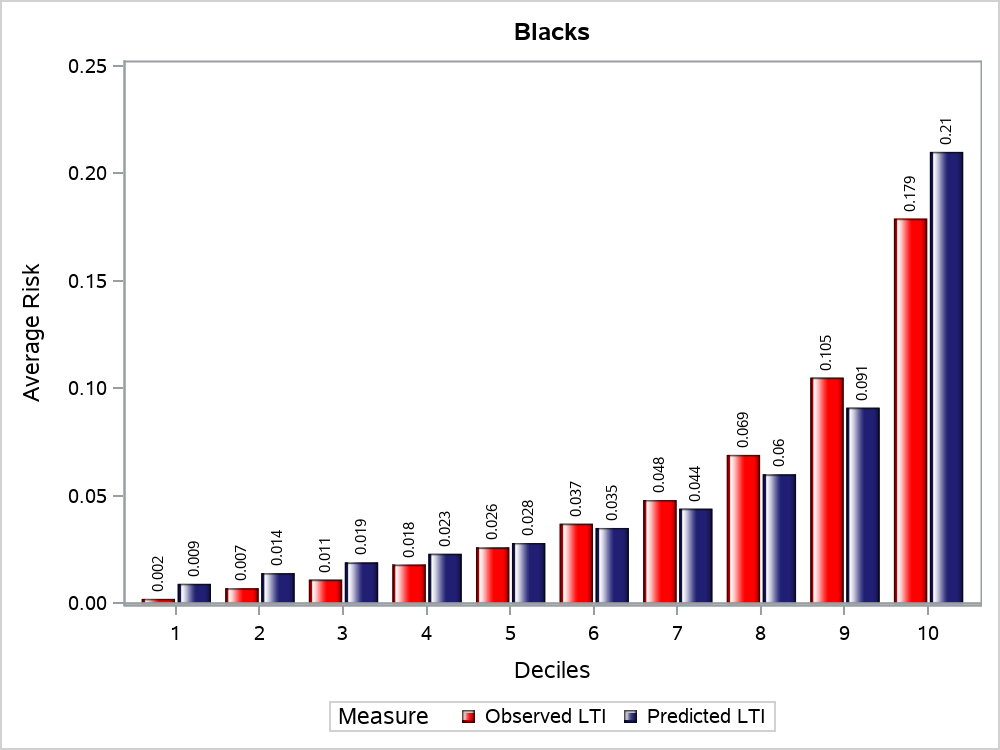


Common Risk


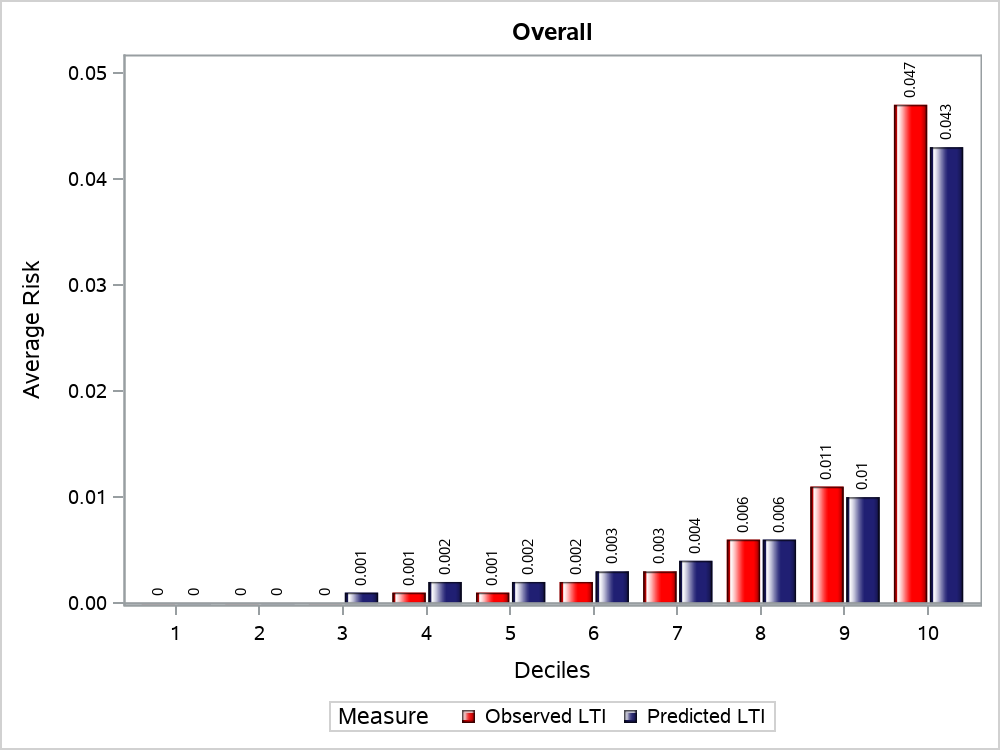

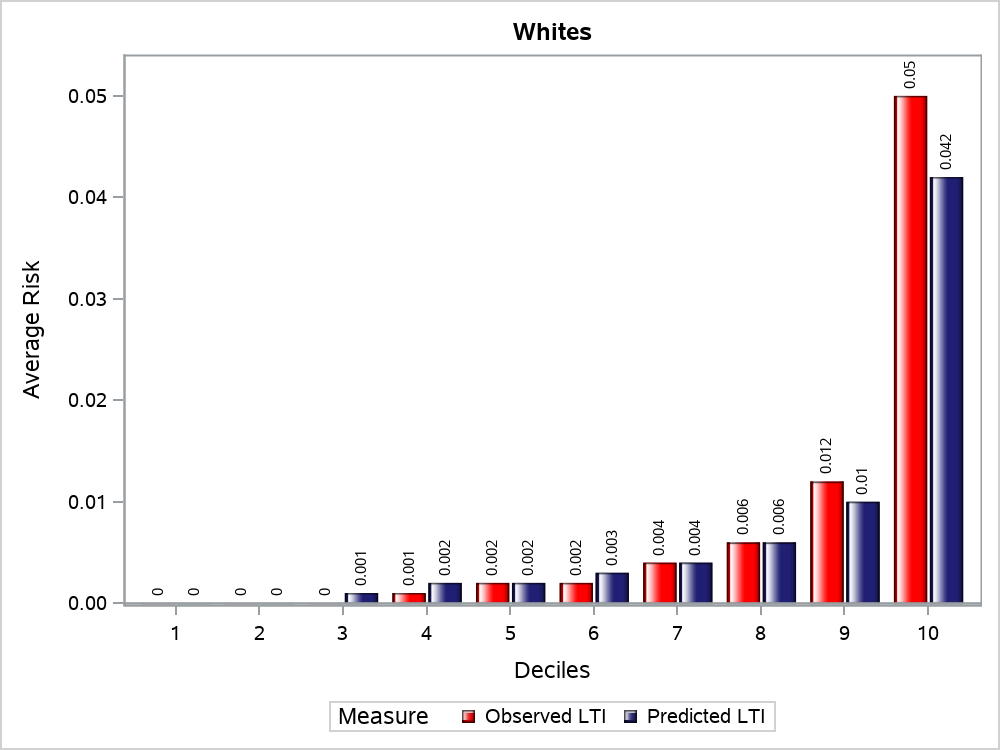

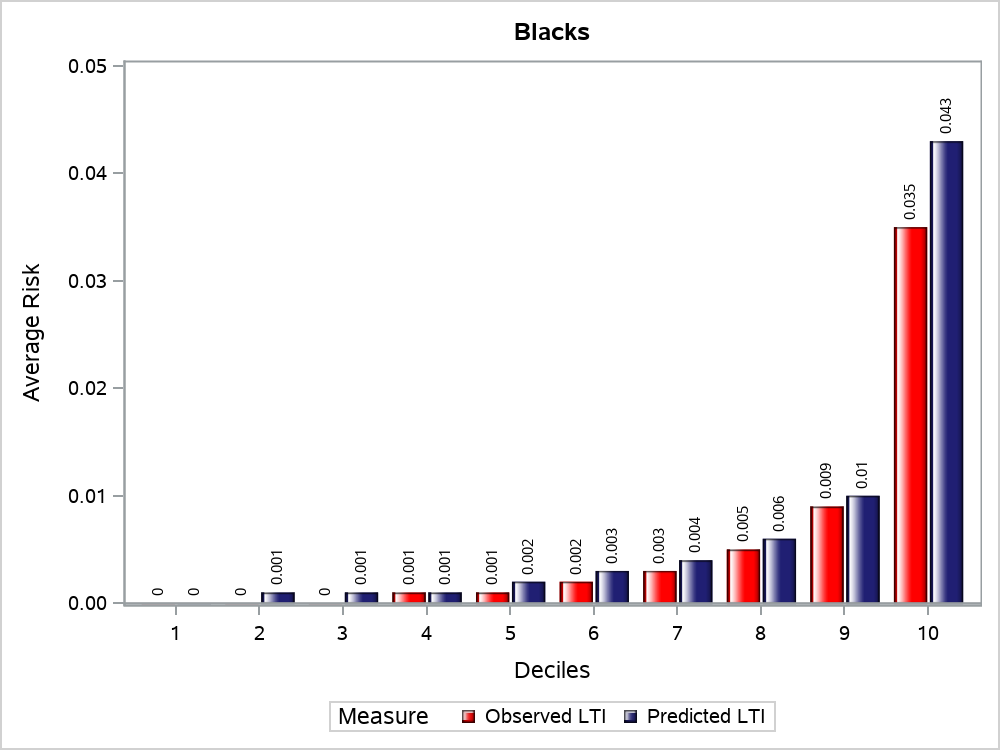


eTable 6. Differences between Logistic and Probit Observed and Predicted Risk by Decile

Elevated Risk

|  | **Overall** | | **White** | | **Black** | |
| --- | --- | --- | --- | --- | --- | --- |
| **decile** | **Logistic** | **Probit** | **Logistic** | **Probit** | **Logistic** | **Probit** |
| 1 | -0.535% | -0.478% | -0.057% | -0.462% | -0.677% | -0.606% |
| 2 | -0.692% | -0.642% | -0.050% | -0.625% | -0.736% | -0.657% |
| 3 | -0.640% | -0.582% | -0.058% | -0.539% | -0.803% | -0.552% |
| 4 | -0.538% | -0.243% | -0.295% | -0.183% | -0.543% | -0.430% |
| 5 | -0.263% | -0.087% | -0.176% | -0.050% | -0.266% | -0.180% |
| 6 | 0.114% | 0.227% | -0.113% | 0.273% | 0.174% | -0.295% |
| 7 | 0.759% | 0.598% | 0.161% | 0.857% | 0.395% | 0.018% |
| 8 | 1.283% | 1.269% | 0.014% | 1.456% | 0.926% | 0.206% |
| 9 | 1.979% | 1.859% | 0.120% | 2.050% | 1.352% | 1.422% |
| 10 | -1.469% | -1.850% | 0.381% | -1.242% | -3.128% | -3.101% |

Common Risk

|  | **Overall** | | **White** | | **Black** | |
| --- | --- | --- | --- | --- | --- | --- |
| **Decile** | **Logistic** | **Probit** | **Logistic** | **Probit** | **Logistic** | **Probit** |
| 1 | -0.011% | 0.001% | -0.012% | 0.002% | -0.017% | -0.008% |
| 2 | -0.030% | -0.009% | -0.021% | -0.008% | -0.043% | -0.022% |
| 3 | -0.052% | -0.027% | -0.025% | -0.018% | -0.075% | -0.051% |
| 4 | -0.069% | -0.042% | -0.027% | -0.036% | -0.087% | -0.066% |
| 5 | -0.088% | -0.071% | -0.017% | -0.062% | -0.119% | -0.082% |
| 6 | -0.094% | -0.079% | -0.015% | -0.072% | -0.094% | -0.099% |
| 7 | -0.096% | -0.068% | -0.028% | -0.045% | -0.116% | -0.169% |
| 8 | -0.047% | -0.002% | -0.045% | 0.055% | -0.119% | -0.147% |
| 9 | 0.095% | 0.140% | -0.045% | 0.234% | -0.054% | -0.165% |
| 10 | 0.393% | 0.147% | 0.246% | 0.452% | -0.786% | -0.821% |

# **S2: Reporting checklist for prediction model development/validation.**

Based on the TRIPOD guidelines

|  |  | Reporting Item | Page Number |
| --- | --- | --- | --- |
| Title |  |  |  |
|  | [#1](https://www.goodreports.org/reporting-checklists/tripod/info/#1) | Identify the study as developing and / or validating a multivariable prediction model, the target population, and the outcome to be predicted. | 5 |
| Abstract |  |  |  |
|  | [#2](https://www.goodreports.org/reporting-checklists/tripod/info/#2) | Provide a summary of objectives, study design, setting, participants, sample size, predictors, outcome, statistical analysis, results, and conclusions. | 3 |
| Introduction |  |  |  |
|  | [#3a](https://www.goodreports.org/reporting-checklists/tripod/info/#3a) | Explain the medical context (including whether diagnostic or prognostic) and rationale for developing or validating the multivariable prediction model, including references to existing models. | 5-6 |
|  | [#3b](https://www.goodreports.org/reporting-checklists/tripod/info/#3b) | Specify the objectives, including whether the study describes the development or validation of the model or both. | 6 |
| Methods |  |  |  |
| Source of data | [#4a](https://www.goodreports.org/reporting-checklists/tripod/info/#4a) | Describe the study design or source of data (e.g., randomized trial, cohort, or registry data), separately for the development and validation data sets, if applicable. | 7 |
| Source of data | [#4b](https://www.goodreports.org/reporting-checklists/tripod/info/#4b) | Specify the key study dates, including start of accrual; end of accrual; and, if applicable, end of follow-up. | 7 |
| Participants | [#5a](https://www.goodreports.org/reporting-checklists/tripod/info/#5a) | Specify key elements of the study setting (e.g., primary care, secondary care, general population) including number and location of centers. | 7 |
| Participants | [#5b](https://www.goodreports.org/reporting-checklists/tripod/info/#5b) | Describe eligibility criteria for participants. | 6 |
| Participants | [#5c](https://www.goodreports.org/reporting-checklists/tripod/info/#5c) | Give details of treatments received, if relevant | 5 |
| Outcome | [#6a](https://www.goodreports.org/reporting-checklists/tripod/info/#6a) | Clearly define the outcome that is predicted by the prediction model, including how and when assessed. | 7 |
| Outcome | [#6b](https://www.goodreports.org/reporting-checklists/tripod/info/#6b) | Report any actions to blind assessment of the outcome to be predicted. | N/A |
| Predictors | [#7a](https://www.goodreports.org/reporting-checklists/tripod/info/#7a) | Clearly define all predictors used in developing or validating the multivariable prediction model, including how and when they were measured | 8 |
| Predictors | [#7b](https://www.goodreports.org/reporting-checklists/tripod/info/#7b) | Report any actions to blind assessment of predictors for the outcome and other predictors. | N/A |
| Sample size | [#8](https://www.goodreports.org/reporting-checklists/tripod/info/#8) | Explain how the study size was arrived at. | 7 |
| Missing data | [#9](https://www.goodreports.org/reporting-checklists/tripod/info/#9) | Describe how missing data were handled (e.g., complete-case analysis, single imputation, multiple imputation) with details of any imputation method. | 11 |
| Statistical analysis methods | [#10a](https://www.goodreports.org/reporting-checklists/tripod/info/#10a) | If you are developing a prediction model describe how predictors were handled in the analyses. | 10 |
| Statistical analysis methods | [#10b](https://www.goodreports.org/reporting-checklists/tripod/info/#10b) | If you are developing a prediction model, specify type of model, all model-building procedures (including any predictor selection), and method for internal validation. | 9-10 |
| Statistical analysis methods | [#10c](https://www.goodreports.org/reporting-checklists/tripod/info/#10c) | If you are validating a prediction model, describe how the predictions were calculated. | N/A |
| Statistical analysis methods | [#10d](https://www.goodreports.org/reporting-checklists/tripod/info/#10d) | Specify all measures used to assess model performance and, if relevant, to compare multiple models. | 9 |
| Statistical analysis methods | [#10e](https://www.goodreports.org/reporting-checklists/tripod/info/#10e) | If you are validating a prediction model, describe any model updating (e.g., recalibration) arising from the validation, if done | N/A |
| Risk groups | [#11](https://www.goodreports.org/reporting-checklists/tripod/info/#11) | Provide details on how risk groups were created, if done. | 9-10 |
| Development vs. validation | [#12](https://www.goodreports.org/reporting-checklists/tripod/info/#12) | For validation, identify any differences from the development data in setting, eligibility criteria, outcome, and predictors. | N/A |
| Results |  |  |  |
| Participants | [#13a](https://www.goodreports.org/reporting-checklists/tripod/info/#13a) | Describe the flow of participants through the study, including the number of participants with and without the outcome and, if applicable, a summary of the follow-up time. A diagram may be helpful. | Figure S1 |
| Participants | [#13b](https://www.goodreports.org/reporting-checklists/tripod/info/#13b) | Describe the characteristics of the participants (basic demographics, clinical features, available predictors), including the number of participants with missing data for predictors and outcome. | Table 1 |
| Participants | [#13c](https://www.goodreports.org/reporting-checklists/tripod/info/#13c) | For validation, show a comparison with the development data of the distribution of important variables (demographics, predictors and outcome). | N/A |
| Model development | [#14a](https://www.goodreports.org/reporting-checklists/tripod/info/#14a) | If developing a model, specify the number of participants and outcome events in each analysis. | 12 |
| Model development | [#14b](https://www.goodreports.org/reporting-checklists/tripod/info/#14b) | If developing a model, report the unadjusted association, if calculated between each candidate predictor and outcome. | Table 1 |
| Model specification | [#15a](https://www.goodreports.org/reporting-checklists/tripod/info/#15a) | If developing a model, present the full prediction model to allow predictions for individuals (i.e., all regression coefficients, and model intercept or baseline survival at a given time point). | Table S2 |
| Model specification | [#15b](https://www.goodreports.org/reporting-checklists/tripod/info/#15b) | If developing a prediction model, explain how to the use it. | 14 |
| Model performance | [#16](https://www.goodreports.org/reporting-checklists/tripod/info/#16) | Report performance measures (with CIs) for the prediction model. | 13, Table S1 |
| Model-updating | [#17](https://www.goodreports.org/reporting-checklists/tripod/info/#17) | If validating a model, report the results from any model updating, if done (i.e., model specification, model performance). | N/A |
| Discussion |  |  |  |
| Limitations | [#18](https://www.goodreports.org/reporting-checklists/tripod/info/#18) | Discuss any limitations of the study (such as nonrepresentative sample, few events per predictor, missing data). | 17 |
| Interpretation | [#19a](https://www.goodreports.org/reporting-checklists/tripod/info/#19a) | For validation, discuss the results with reference to performance in the development data, and any other validation data | 15 |
| Interpretation | [#19b](https://www.goodreports.org/reporting-checklists/tripod/info/#19b) | Give an overall interpretation of the results, considering objectives, limitations, results from similar studies, and other relevant evidence. | 16 |
| Implications | [#20](https://www.goodreports.org/reporting-checklists/tripod/info/#20) | Discuss the potential clinical use of the model and implications for future research | 17 |
| Other information |  |  |  |
| Supplementary information | [#21](https://www.goodreports.org/reporting-checklists/tripod/info/#21) | Provide information about the availability of supplementary resources, such as study protocol, Web calculator, and data sets. | S1 |
| Funding | [#22](https://www.goodreports.org/reporting-checklists/tripod/info/#22) | Give the source of funding and the role of the funders for the present study. | 1,19 |

Notes:

- 16: 13, Table S1 The TRIPOD checklist is distributed under the terms of the Creative Commons Attribution License CC-BY. This checklist was completed on 04. July 2024 using <https://www.goodreports.org/>, a tool made by the [EQUATOR Network](https://www.equator-network.org) in collaboration with [Penelope.ai](https://www.penelope.ai)
